# Augmented Reality, a Review of a Way to Represent and Manipulate 3D Chemical Structures

**DOI:** 10.1021/acs.jcim.1c01255

**Published:** 2022-04-04

**Authors:** Alba Fombona-Pascual, Javier Fombona, Rubén Vicente

**Affiliations:** † Organic and Inorganic Chemistry Department, University of Oviedo, Av. Julian Clavería, Oviedo 33006, Spain; ‡ Education Sciences Department, University of Oviedo, C. Aniceto Sela, Oviedo 33005, Spain

## Abstract

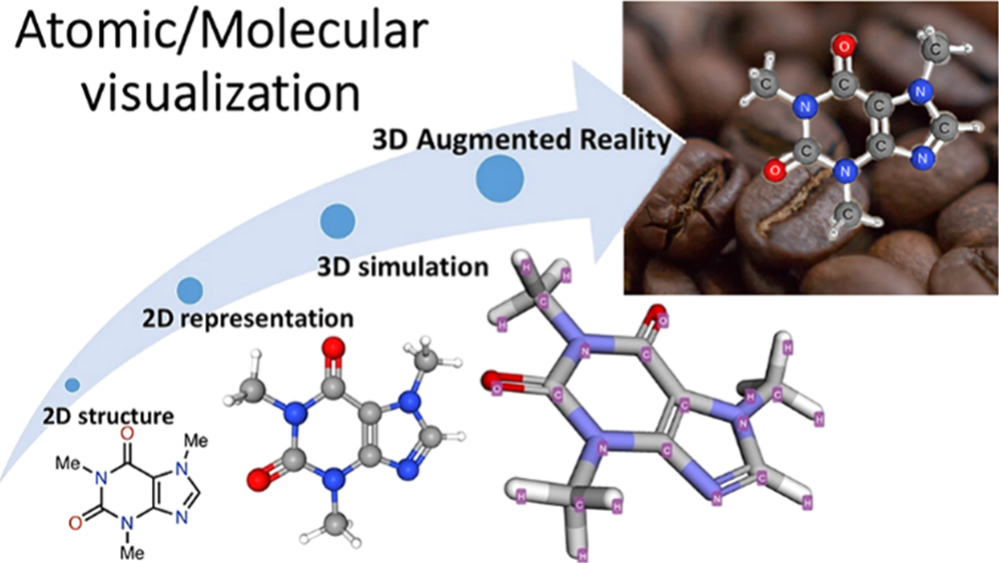

Augmented reality (AR) is a mixed technology that superimposes three-dimensional (3D) digital data onto an image of reality. This technology enables users to represent and manipulate 3D chemical structures. In spite of its potential, the use of these tools in chemistry is still scarce. The aim of this work is to identify the real situation of AR developments and its potential for 3D visualization of molecules. A descriptive analysis of a selection of 143 research publications (extracted from Web of Science between 2018 and 2020) highlights some significant AR examples that had been implemented in chemistry, in both education and research environments. Although the traditional 2D screen visualization is still preferred when teaching chemistry, the application of AR in early education has shown potential to facilitate the understanding and visualization of chemical structures. The increasing connectivity of the AR technology to web platforms and scientific networks should translate into new opportunities for teaching and learning strategies.

## Introduction

Chemical structures are inherently three-dimensional (3D), yet their representations are generally constrained to 2D space, especially when depicted in paper or on screen. For instance, relevant features typically covered by stereochemistry, crystallography, or molecular symmetry are normally approached only in the 2D form. As a result, making viable and graphically accurate reproductions of complex chemical structures^1^ is often challenging for chemistry students even at higher education levels, for example, performing complex quantum mechanics.^2^

**Figure 1:**
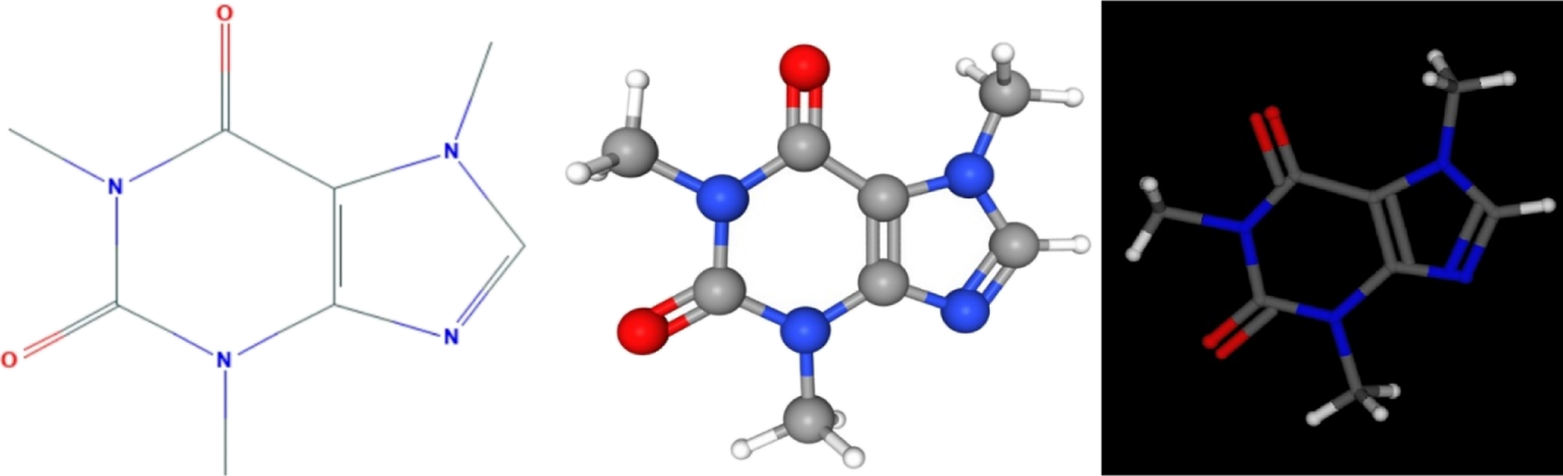
Archetype representation of caffeine (1,3,7-trimethylxanthine) (left: chemical structure from National Institute of Standards and Technology; middle: screenshot from PubChem online software; and right: screenshot from Molecule3D 3.9 offline application for smartphones).

Considering these issues, technological complements and visualization supported through simulation or digital representation are necessary.^3,4^ In this sense, descriptive resources ranging from electron microscopy images to drawings, scale models, or even analog videos^5,6^ have been deployed with different success. Nevertheless, these resources still require considerable capacity for visual and spatial thinking.^7^ Thus, it is common to find presentations of 3D information using 2D projections in chemistry research even though this undermines true representation of its characteristics (Figure 1).^8,9^

Augmented reality (AR) is a mixed technology that superimposes a 3D digital image onto reality. Normally, the representation of 3D models using AR is done with a video camera on a computer screen, smartphone, or head-mounted display (HMD) with semitransparent glasses, such as HoloLens, Microsoft.^10^ AR usually produces a surprising visual impact for the user, who easily perceives the spatial dimensionality of the 3D digital images emerging and moving superimposed on real figures. This surprising trait is being used for marketing purposes,^11^ including the feasibility to produce 3D books.^12^ In spite of their potential benefits, the use of AR still remains underdeveloped in chemistry, particularly in an academic environment.^13^

AR can significantly enhance human performance, especially in the educational field.^14^ This technology seems to be a powerful tool to facilitate the acquisition of competence in spatial visualization skills^15,16^ and could also serve as a source of motivation for learning, although little information on its impact is available. This review collects and analyzes the use of AR in chemistry with the aim of triggering the implementation of this appealing technology. 3D representations can favor the process of dissemination of the research results, and these images have an impact on other people interested in understanding the phenomenon investigated. The results of research are transferred to society through different ways, and education constitutes a suitable platform to apply innovations systematically. In this sense, the use of AR in education could serve as an important tool to facilitate the incorporation of innovations in a more efficient manner. Specific features of AR, such as visualization and 3D representation capabilities, and its simplicity are relevant to education because it serves as a motivation and resulted in better results in the assessment.

## Previous Research on Chemistry Education and AR

At the beginning of the 1990s, Rosenberg introduced the term “AR”.^17^ In the first decade of the 21st century, this tool was applied in training within laboratories working effectively with virtual instruments.^18^ There are reviews of scientific literature that describe how AR technology itself works,^19,20^ and the speed of technological evolution means that the analyses of these topics become rapidly outdated.^21−24^ For instance, AR research delves into procedures to trigger digitally stored information and its superposition on real images,^25^ detecting the spatial position and pose of user’s fingers.^19^

The first reports of AR regarding the teaching of chemistry appear in the 2010s. Most of these reports were published at conferences, introducing new technological designs and the advantages this interaction could bring to the education of chemistry.^26^ For instance, the development of new interfaces called augmented chemistry (AC), where elements can be composed into 3D molecular models,^27^ or the first experiences with HMD HoloLens glasses for AR in inorganic chemistry were disclosed.^28^ Thus, AR could be an interesting solution to the problems related to 3D crystal structures in inorganic chemistry, and undergraduate students might understand these topics, facing symmetry-related problems supported by 3D figures.^29^

Previous reviews of AR in education by scientific literature articles described, in general, a positive impact of AR on academic outcomes.^22,24^ In this sense, new hardware developments have also appeared, as well as some proposals describing haptic prototypes developed to work with augmented chemical reactions have also appeared.^30^ AR emerges with other technological advances and new educational strategies, such as gamification.^8,31^ AR and these methodologies seem to change the traditional teachings that introduce educational and collaborative games in chemistry.^32^

AR tends to improve students’ motivation.^23^ Indeed, several studies highlight how this tool with computer-assisted learning improved the results of low-achieving students, as well as triggered a positive attitude.^33^ There are hardly any studies that describe problems generated after the introduction of these technologies. In this way, some students liked to manipulate AR by rotating markers, while many of them preferred to interact with physical models in order to get a feeling of physical contact.^34^

The implementation and incorporation of AR technology, especially at the university level has been growing at a slow pace.^35,36^ In addition, most of the analyses of AR experiences were limited to studies of a general nature, such as organic chemistry^37^ or, in contrast, were used in very specific topics. For instance, various experiences in biochemistry have been described based on these technological developments, such as AR metabolic pathways ARMET,^38^ which allows students to visualize 3D molecular structures and evaluate their own levels of understanding.

More importantly, there is a lack of reviews specifically focused on scientific literature related to AR and chemistry. This new technology needs to be further explored in order to agree on a solid theoretical and practical approach.^16^ Along these lines, it could be a priority to collect the practices of teachers in their experimental laboratories.^16^ Thus, a review of the recent research results published in the scientific literature is essential to provide an overall view of available tools, for implementation in further research and in various educational sectors. The aim of this study is to clarify the main findings from research on AR applied to educational environments in the area of chemistry. This might serve to provide some guidelines to university lecturers for the incorporation of AR technology into chemistry studies. The methodological design of this study focuses on a qualitative descriptive analysis of the impact of AR.

## Methods

### Phase 1: Selection of the Sample of Documents

The scientific database Web of Science Clarivate Analytics (WoS) provided us with relevant research material of proven quality with objective indicators. WoS offers collected data with reliable measures of the impact of the research, including the amount and type of citations. As for type of documents, research results published in books, articles, and congress communications were selected. The cases with the greatest scientific impact are classified within the Journal Citation Report (JCR) level.

Keywords were entered to locate the relevant documents in this repository by applying BIbExcel2016.2.20 software to a set of reiterative terms in the JCR-classified articles; for example, “AR”, “3 dimension visualization”, “3D”, “stereoscopic interface”, “molecular visualization software”, “scientific visualization”, and “immersive analytics”. The terms were linked to “chemistry”, and the search was run to determine which of these terms extracted the most registers related to “advanced atomic/molecular visualization”. The most representative terms generated by this analysis were “AR” and “chemistry”, and the categorization of registers was considered if the terms appeared in the title, abstract, or keywords.

WoS All Databases contain a universe of several million registers from all over the world. In the case of the population of descriptors, the terms “chemistry” and “AR” provided a total of 235 registers (Table 1). Such a high number of registers led us to reduce the sample size by time restriction, for scientific convenience because research on older technology could now be out of date in theoretical and practical terms.

**Table 1:** Research Papers on the WoS

Years	no. of documents with ≪chemistry≫ and ≪augmented reality≫ in the title, abstract, or keywords	
2015	7	
2016	10	
2017	21	
2018	34	
2019	65	
2020	44	
2018 to 2020 (period analyzed)	143	
	article (81)	56.64%
	proceeding (42)	29.37%
	book (3)	2.11%
	other (17)	11.88%
“all years” 1900 to 2020	235	
	article (115)	48.94%
	proceeding (86)	36.60%
	book (5)	2.12%
	other (29)	12.34%

Analysis by date shows that AR had a reduced impact before 2017. Because the inventions from a few years ago seem to be obsolete today, new technology is being reconfigured. Actually, there are not many studies on AR in Chemistry published in high impact journals before those dates. This technology might be renewed because of the augmented capacity of smartphones’ ability to play audio, take pictures, as well as viewing videos. Smartphones and tablets can now be used to perform many tasks previously addressed by desktop computers.^39^ Since then, a significant number of research articles on teaching chemistry using AR have appeared, as indicated by Table 1. It has also been observed that the number of investigations carried out in 2020 declines again, probably due to the global impact of Covid-19 crisis. The overall evolution suggested that analysis of publications for the period 2018–2020 should be sufficiently representative, offering a total of 143 documents fulfilling the search criteria. Most of the documents appeared in scientific journals as research articles, suggesting that it is an emerging phenomenon. In contrast, documents extracted from books are still scarce indicating the lack of revisions of the topic.

### Phase 2: Content Analysis of the Documents

Content analysis was performed on complete documents in the text form downloaded from the WoS platform; this database analysis was coded using tools such as Aquad7 software. The texts were converted into *.rtf format and the concepts were counted together, with the frequency of their appearance and interrelations as the indicator of their strength related to the key terms “chemistry” and “augmented”. There were two stages: (1) verification of whether the title, descriptors, and abstract contained visualization experiences and, if so, the next step of the analysis continued. (2) Analysis and extraction of findings in each document, prioritizing results, discussion, and conclusion. Additionally, a confirmation of whether the text reached a JCR category document was accomplished.

## Research Rigorousness and Limitations

The analysis of research at the JCR level assumes a guarantee of high scientific quality and relevance in these documents. Gathering data for qualitative analysis is difficult yet Aquad7 software allowed us to examine texts accurately. Two external professors have participated in this part of the study. This analysis includes the evaluation of the articles, extracting the related fundamental terms and determining their importance in each text. Thus, we measure its objective relevance by checking its position in the title, within the list of keywords or/and within the abstract. The most important findings were also quantified and these terms were grouped and quantified, giving them special value when they were repeated.

Among the limitations of this research is the fact that there were few previous studies in chemistry of this nature to draw on, although this could be seen as an opportunity to identify new lines in the literature and investigation. Even though this research successfully analyzes all the relevant elements and achieves its goals, this article is only a snapshot of existing research on atomic/molecular representation, and the area needs further and deeper investigation. The number of publications analyzed was limited in comparison with the broad scope of AR technology. In this sense, the present work is certainly exploratory and tries to shed some light on a complex scenario such as AR technology. Accordingly, only reports regarding application and implementation of AR in education, chemistry, have been analyzed.

## Results and Analysis of the Data on JCR Research on Visualization Using AR

The performed analysis of the contents differentiated and contrasted the following themes: (a) two-dimensional atomic/molecular representation software and (b) JCR research on AR.

### Repertoire of 2D Atomic/Molecular Representation Software

First, this study reviewed the software on internet based on a search of the terms “molecular atomic visualization software”; this search yielded 175 applications that possessed a functionality related to this type of 2D representation on a screen, including most of the operative systems. The large number of 2D representation programs ranges from desktop software to others working from a specific web site or cloud software, including open source and licensed commercial software or applications. There are tools for specific areas of Chemistry as well as multidisciplinary areas. This fact makes it difficult to categorize these computer tools given their variability in time. Hence, we opted for an open sample of software available for the users (Table 2).

**Table 2:** Software for Atomic/Molecular Calculation, Visualization, and Manipulation on a 2D Screen, without Stereoscopic Vision (Details of This List Are Provided in the Supporting Information)^a^

-3dmol.js (*)	-Cuemol (*+)	-MolScript (*)
-Abalone	-Discovery Studio	-Molsketch (*+)
-ABINIT (*+)	-Drug Bank (web)	-MoluCAD (*)
-ACD/ChemSketch	-DSSP (*+)	-MolView (*web)
-ACES II (*)	-EGO (*)	-MOPLOT (*+)
-Amsterdam Density Functional ADF	-enCIFer	-MPQC (*)
-Advanced Simulation Library (ASL) (*+)	-ePMV (*+)	-NAMD Molecular visualizations (*+)
-AIMAll (*+)	-EzMol (*web)	-NAMOT (*+)
-AltPDB Protein-viewer-activity (*+)	-FHI-aims (+)	-NAOMI/Unicon (*+)
-AMBER (*)	-Gabedit (*+)	-Newton-X (*)
-Amira	-GASP (*+)	-NGL viewer molecules (*web)
-AMPAC	-Gaussian – Gauss View (*)	-OctaDist (*+)
-AMSOL	-GeNMR (web)	-Octopus (*+)
-Ansys Chemkin-Pro	-Ghemical-GMS (*)	-OpenAtom (*+)
-Ascalaph Designer (*+)	-gOpenMol (*)	-OSRA (*+)
-Atomistic	-GPCR-ModSim (*web)	-PDB Protein Data Bank (*web)
-Autochem	-GRAMM (*)	-Perse Visualizer (*)
-AutoDock (*+)	-GRAMM-X (*web)	-POLYVIEW-3D (*web)
-Avizo	-GRASP	-PovChem (*)
-Avogadro (*+)	-GROMACS (*+)	-PQS 3D stereo systems
-Babel (*+)	-GROMOS (*+)	-Prosat+ (*web)
-BALL (*)	-HBPLUS (*)	-Protein Explorer (*web)
-BigDFT (*)	-HINT!	-PubChem 3D (*)
-BioBlender (*+)	-ICM Chemist/Molsoft	-PV-JavaScript Protein Viewer (*+)
-BIOVIA	-ICM-Browser/Molsoft	-PyMol
-Biskit (*)	-IcmJS/Molsoft	-Python Molecular Viewer PMV (*+)
-BKChem (*)	-IMol (*)	-Q-Chem
-BRAGI (*)	-Insight II	-Quantum ESPRESSO (*)
-CADPAC (*)	-ISIS Draw (*)	-QUEST (*)
-Cambridge Structural Database (CSD) (*)	-JChemPaint (*)	-QuteMol (*+)
-Cantera (*)	-Jmol/JSmol (*+)	-RasMol (*+)
-Car–Parrinello molecular dynamics (*)	-Jolecule Protein viewer (*web)	-Raster3D (*+)
-CASINO (*+)	-JSME/JME Molecule Editor (*+)	-RasTop (*+)
-CASTEP (*)	-Kinemage, MAGE &; King (*)	-RCSB MBT Viewers (*+)
-CAVEAT (*)	-LigPlot (*+)	-Ribbons (*web)
-CCDVault (web)	-LiSiCA (*+)	-RINalyzer (*)
-CCP4MG (*)	-Loopy (*)	-Rpluto (*+)
-CHARMM (*)	-Luscus (*+)	-SAMSON
-Chem 4-d (*)	-MacroModel	-Scigress
-Chemcraft (*)	-MADNESS (*+)	-ShelXle (*+)
-Chemical Shift Index (*)	-Marvin (*)	-SIESTA (*+)
-Chemical WorkBench (*)	-MC-SYM (*web)	-Spartan (+)
-Chemicalize (web)	-MDL Chime (*)	-Swiss PDB viewer (*)
-Chemistry Development Kit (*)	-Mercury Crystal Structure Visual	-SYBYL (*+)
-ChemOffice/ChemDraw (*)	-MGLTools (Python &; AutoDock) (*+)	-TeraChem (+)
-Chemsketch	-MODELLER (*+)	-Tinker Molecular Modeling (*+)
-ChemSpider (*web)	-Moil (*+)	-Ugene (*+)
-ChemVLab+ (*web)	-Mol2Mol (*)	-VASPMO
-ChemWindow (*	-MOLCAS (*)	-VIDA (*+)
-CHIME (*)	-Molconn-z (*+)	-Vienna Ab initio Simulation
-Chimera (*web)	-Molden (*+)	-Vibeplot (*+)
-Chemitorium (*+)	-Moldraw (*)	-Virtual Chemistry 3D (*web)
-Cn3D (*)	-Molecular Operating Environment	-Visual Molecular DynamicVMD (*+)
-Computational Center MacromolecularS.(*+)	-Molecular Workbench-Concord	-WebLab Viewer (*)
-CONQUEST	-Molegro Virtual Docker	-XDrawChem (*+)
-Coot (*+)	-MOLEKEL (*+)	-Yasara (*+)
-CP2K (*+)	-MOLGEN (*web)	-Zeus (*)
-CrystalMaker	-MolPOV2 (*)	
-CS23D (*web)	-MolPro	

a (+)Open source, (web) visualization on website, and (*) free software; some of them are free for academic and non-commercial use.

Programs for the 2D visualization of chemical structures on screens collected in Table 2 are used for multiple purposes in various disciplines, and a brief description of them is included in the Supporting Information. The majority of those tools allow the user to build and/or analyze the geometry of the molecular systems. In addition to this, some of them (i.e., Avogadro, Molden, or GaussView) incorporate the realization of MM (molecular mechanics) calculations, the visualization of molecular orbitals and molecular surfaces, and the animation of normal modes of vibration or the possibility to perform conformational analysis. Programs providing chemical structures based on density functional theory or ab initio calculations are also included.^40^

However, it is difficult to make a universal classification, and we found that users combine several programs simultaneously. For instance, there are 3D molecular simulation experiments that use software such as PyMOL with Python language programing, and open-source programing language libraries to create large multidimensional vectors and matrices, such as NumPy.^41^ Applications based on Web browsers such as Iview and Jmol, a Java-based molecular visualization tool, are also employed to simulate 3D molecules on a 2D computer screen.^8^ Moreover, interesting experiences of 3D model generation with open-source optical structure recognition application (OSRA) together with the simplified molecular input line-entry system and Jmol are commonly used. We observed that users handle various computer tools in a complementary way. This combination of software is capable of narrowing the performance gap between students with different levels of spatial perception abilities.^42^

As already indicated, there is an abundance of specific software for smartphones and tablets. APP is the generic name of these specialized programs for mobile devices. Google Play repositories for Android and the Apple APP store for IOs Apple have hundreds of such tools. Many of them are offered in both repositories, as is the case of MoleculAR.^43^ These APPs have only emerged recently and are normally free or low-cost, with underpowered molecular visualization. Researchers who have analyzed these APPs indicated that their applications are still mainly restricted to simple molecular structure visualization.^44^

### Research on AR

There is a set of investigations on the 3D representation of the atomic/molecular structure using AR or mixed reality technology. For a basic operation, AR runs with computing equipment along with a device capable of capturing images, such as a webcam, and a specific program to superimpose digital objects on the image of the reality across the user’s screen. Most recently, these APPs are increasing their presence on smartphones,^3,14,43−46^ and users can view chemistry concepts, interact with them by hand, and learn through a self-directed learning experience.^47^

**Figure 2:**
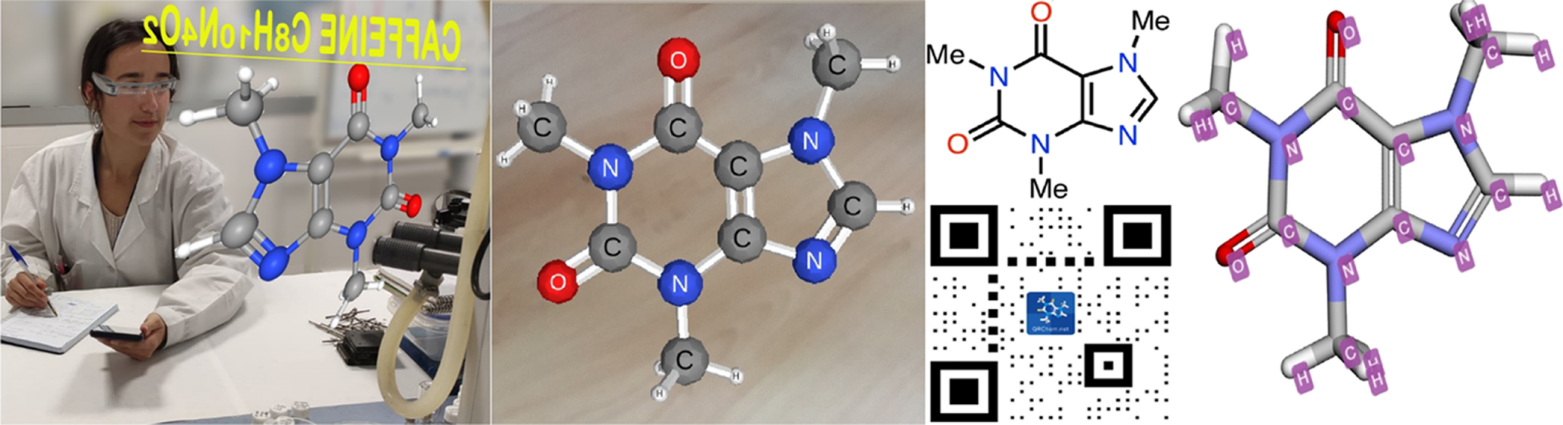
Examples of AR visualization of the molecule caffeine (1,3,7-trimethylpurine-2,6-dione) C_8_H_10_N_4_O_2_. First, shot of a user handling the molecule, with AR Moverio BT-300 AR Glasses; second, screenshot of a smartphone with the AR picture of this molecule with model AR1.3, software APP for smartphone offline; third and fourth, 2D structure and QR code generated with the AR QRChem software for PC online (QRChem.net); and fifth, screenshot of the 3D simulation resulting with QRChem software.

There are two kinds of 3D technologies; AR, which adds digital elements in real-world, and virtual reality (VR), which makes an entire digital world around the user in a stereoscopic vision.^48^ Research on AR also emphasizes the emergence of increasingly powerful hardware such as HMD equipment with semitransparent glasses to allow the user to view 3D digital data overlapping reality (Figure 2). These HMD devices are providing less-immersive experiences than VR,^49^ but some of them have the capacity to follow the user’s eye movements.

Currently, the technological evolution of AR allows interactions with objects through digital monitoring of the user’s hands, in a similar way as it happens in the VR technology.^50,51^ Several researchers have compared these HoloLens AR glasses with visualization experiences on 2D computer screens or laptops, which shows that traditional equipment is faster and more precise.^52^ That is why some authors have demonstrated the shortcomings of the AR technology and why resources for visualizing chemical reactions are limited to static models and preprogramed animations.^47^ In this sense, the lack of user interactivity with real experiences could be an important aspect of teaching, and this cognitive skill is crucial to the understanding of chemistry as a subject.^47^

Other investigations have examined the potential of programs, such as ARChemEx, ARKimia Kit, AC,^53^ Vuforia,^54,55^ and ARchemy.^56^ The specific use of AR with positive repercussions on the field of chemical education is described in various investigations,^57−59^ especially at lower levels of training.^60^ Indeed, they analyzed the outcomes of understanding the phenomena in the short and medium term. Students were positive to this experience, with a willingness to become active learners. In particular, application in experimental courses showed its utility to teach while minimizing the use of chemicals, reducing handling risks and environmental impact.^54^

**Figure 3:**
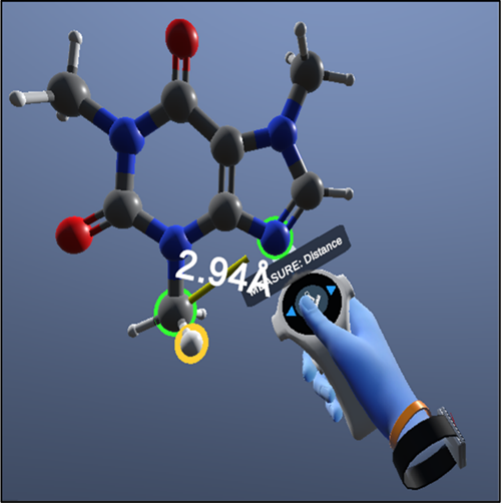
Example of VR visualization of the 3D molecule, caffeine (1,3,7-trimethylxanthine) C_8_H_10_N_4_O_2_. The avatar/user’s hand is measuring the distance between two atoms. Picture saved from oculus glasses hardware and Nanome 2021 Inc. VR software. There are really two images with small differences, each image is projected onto one eye, this produces stereoscopic vision.

Research on AR stressed its simplicity and flexibility when compared to VR (Figure 3). Because VR has a complex technology, it requires more sophisticated software to handle. Furthermore, VR users are isolated by HMD devices, completely immersed in a virtual environment, being cut off from their surroundings.^61^ Having said that HMD headsets are evolving their portability, and the improvements try to overcome the motion sickness and the sense of isolation.^48^

**Figure 4:**
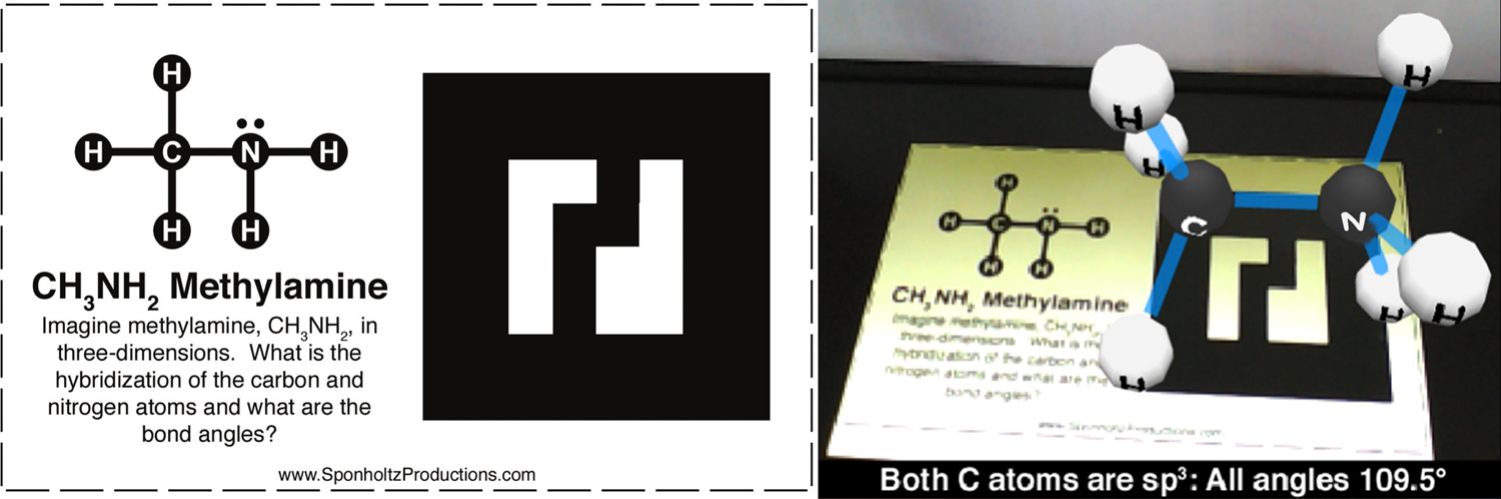
Example of AR visualization of a methylamine molecule over the camera-view when users focus on the AR specific marker, a card/key-picture to capture through the webcam and the AR software for PC offline (SponholtzProductions). The screenshot with the AR card with the methylamine molecule stored that over imposes reality in a 3D simulation.

AR is linked to real elements of the reality and the uploading of digital data for visualization is key. Normally, users can print a card-marker or employ a camera shooting pattern and launch their own interactions (Figure 4). This allows viewing the digital file on top of an image captured with a video camera.

These card-markers usually appear in printed form in books and magazines or can be executed from websites and function on normal computers. For the fabrication of these self-made shooting patterns, undergraduate students download and print the marker. Then, pointing a smartphone or a tablet toward this pattern, a previously stored digital image, like a molecule, is superimposed. A specific benefit lies in the illusion of moving simultaneously with the projection, and the possibility of interaction with the image, including rotation and translation of the chemical structures along 3-axis, as well as enlarging and reducing their size as required. The AR task ensures a personal pace for understanding complicated systems^46^ and provides an individual approach for enhanced learning. Importantly, undergraduate students enjoy using these methods.^59^

Work on AR visualization can also be driven by the use of quick response (QR) codes (Figure 2), as examined in a number of works on structural 3D models.^61^ The visualization software of 3D can be sophisticated for manipulating and visualizing a model in almost infinite ways; however, the procedure with QR codes is straightforward in representing structural data. This can be used in lectures or seminars by projecting/presenting these codes, and the audience can directly reproduce the 3D structure on their mobile device. The application of this strategy is not only limited to represent molecules, including large ones such as proteins, but also allows the elaboration of 3D data sets from the results from spectrometry experiments to map interaction partners.^62^

All this research underlines the fact that AR techniques can be used over a period of time longer than VR. Moreover, the AR technology allows users to work in parallel by taking notes or handle other documents in another screen.^63^ Once again, AR was found to be motivating for learners,^64^ providing valuable digital 3D information for visualizing discrete molecules as well as their changes during chemical transformations.^65^

As stated above, the documents analyzed in this review clearly emphasized that this technology is not restricted to visualizing structures but enables these elements to be controlled and manipulated.^9^ In this sense, students showed a marked preference for more tactile AR models, providing the user a greater control over molecular manipulations.^4,9^ The majority of experiences with AR have been focused on early education because of the simplicity for its implementation. For instance, AR was employed to study redox reactions using Vuforia SDK and Unity3d^55^ or in molecular geometry.^66,67^ Improvements in performance were noted in preuniversity students when working with HP Reveal software to identify organic compound formulas and nomenclatures, chemical bonds, and intermolecular forces.^37^ At the university level, studies on chemical kinetics, chromatography, or the Krebs cycle reported improvements in the understanding of complex concepts, as long as they are visually presented.^1,68^ Noticeable improvements of the assimilation of concepts such as the understanding of molecular symmetry with diverse structural geometries^7^ have been reported by using the molecular symmetry alternate conception test, MACT.

Nevertheless, AR research in education does not only describe successful experiences, some studies did not observe beneficial effects of this technology.^69^ This line includes the result of the activity in virtual laboratories (VL), and these results were similar to those obtained in real laboratories with traditional resources.^4^ Other research showed improvements in learning outcomes^70^ and understanding in practical chemistry classes.^71^ In spite of these discrepancies, all these features together justify the growing use of AR in education and research.

In general, some key findings of AR can be described so far:

- AR is a relatively simple technology with most of the documented experiences focused on an early education stage. This technology can help to visualize 3D molecules in an effective and efficient way.^42^ Computer tools are commonly used, while APPs and software for smartphones are still used for more simple structures.^44^
- AR is motivating for the students,^58,64,72^ who enjoy using AR.^73^
- AR might be helpful to minimize costs and risks in experimental courses.^54^
- Improvements in the educational performance with AR are still not obvious.^68,69^
- 3D simulations and interactive atomic/molecular representation software are widely, effectively used on 2D screens of the traditional computer terminal, in chemistry classes of all levels. AR has problems for its systematic incorporation in educational environments.^74^ In this sense, these technologies are novel and lack a proven track record of effectiveness. Moreover, the required tools, such as HoloLens glasses, need a high-performance computing to display 3D motion images, and it can be expensive for students.

## Outlook on Future Research

Current 3D chemical representations and manipulations are growing across all stages in chemistry,^75^ in spite of the difficulties in developing molecular software fulfilling rigorous scientific criteria. Regarding educational environments, it seems advisable to incorporate gradually the AR at early stages of chemistry studies according to a number of studies supporting its effectiveness. For instance, AR visualizations appear as a valuable complementary tool to learn key concepts such as chirality, stereochemistry, coordination of metal complexes, and biomacromolecular structures^6^ or for more advanced topics such as exploration of molecular surfaces, geometries, transition states, or intermolecular interactions in biomolecules, among others. Additionally, AR might be an interesting complement to assist laboratory courses through the use of digital replicas. The trend observed suggests that many of the next learning scenarios will be virtual workspaces.^4^ Nevertheless, the technological literacy of chemistry teachers must be significantly increased,^13^ and teachers need to review the most appropriate learning methodologies to apply AR effectively in education.^16^

AR technology makes use of software for chemical visualization: online programs, PC software, and APPs for smartphones. Additionally, there is an AR markup language (ARML), which is being used in XML grammar and software development kits (SDK) for simple AR environments. The biggest change driven by AR will be a human interaction with reality, including digital component in this process. In this way, new gestural patterns are being incorporated to facilitate the interaction and recognition of user gestures without the need of an intermediate interface, such as a camera making an eye-tracking.^76^ The emerging technologies still require rigorous academic and scientific analysis,^16^ particularly in the field of education.^58,72^

People use their mobile phones in countless ways that are becoming a part of the educational activity of both students and teachers. Currently, smartphones have some limitations to operate with AR software, but these APPs will have a more effective and complex use in chemistry. This will be combined with artificial intelligence, the big data, the networks, and global collaboration.^77^

Investigations that analyze the research cooperative networks are of particular interest, and some of these experiences are supported by decentralized and interconnected databases.^77^ The science needs increasingly collaborative work to solve global challenges. In this sense, many researchers are starting to pursue open-source software development collaborative projects to tackle a globally accessible and effective chemistry teaching.^2,20^

It is also interesting to know new sophisticated virtual environments that simulate reality,^78^ for instance, in complex networks of chemical reactions based on huge quantities of data and vast quantum and chemical robotized explorations. The 3D scenes digitally recreated are becoming powerful tools for exploring molecule and protein energy landscapes.^78^

The interaction of reality data with digital information is the foundation of the machine learning, where computers can learn from data without being programed by people,^79,80^ and this augments conventional physics-based approaches in computational research.^80^ This new technology infuses robustness into the molecular models, and it is bound to become a mainstream tool in chemical research.^2^ Hence, we encourage more research work into how all these possibilities could have an educational utility.

## Conclusions

The results provide a snapshot of current research on advanced atomic/molecular representation. The AR technology helps to visualize complex 3D environments in chemistry that today are done only in the laboratory, for example, to understand chemical reaction mechanisms and their evolution in an optimization or a reaction path. AR allows analyzing different molecular characteristics, such as structures, bond distances, dihedral angles, geometry of a system, calculation of electrostatic potentials and vibrational modes, and so forth. These types of visualizations are efficient showing 3D molecules with stereocenters, as they could display an amino acid, a chiral catalyst, or processes where stereochemistry is relevant.

The main research findings reveal discrepancies regarding the effectiveness of this technology, especially when applied to higher education. Thus, the representation on a bidimensional screen is used in chemistry classes at all educational levels, whereas the AR technology has a further practice among initial students. In this sense, smartphone applications focus the content primarily on the visualization of simple molecular structures, while more advanced users prefer traditional atomic representation. This fact is justified because the latter equipment is faster and more precise, especially when they combine several programs simultaneously in order to see and to understand complex molecular interactions.

Simple AR software is now becoming widely available, and the students usually have smartphones capable of showing these pictures. AR is effective when students are relatively new to the laboratory environment, and when the user is working on complex processes and need assistance provided by additional digital information. These graphic tools are not critical to the analysis of chemical reactions, but might help the 3D perception, which makes it more interesting for students, increasing their stimulation to understand chemistry. Therefore, it seems advisable to incorporate this technology into early chemistry education.

The publications analyzed concur in that implementation of the technologies that simulate this reality cannot replace in-person experiences but they can complement them. This fact is especially relevant when real experiments are impeded by physical obstacles. In this respect, these digital technologies should contribute to the development of an effective online accessible education.

To conclude, this analysis can contribute to the identification of potential practical references for teachers and academics, as well as for researchers, who are designing the future smart educational environment. Finally, inclusion of scientific innovation and these technologies as complementary tools might result in a symbiotic effect for teaching chemistry.

## Supporting Information Available

Data and software availability; original list with 315 applications for visualization that we have analyzed to select 175 applications; and analysis and brief description of the software for on-screen visualization and manipulation (PDF)

## Abbreviations

2D: two-dimensional
3D: three-dimensional
AR: augmented reality
ARML: AR markup language
HMD: head-mounted display
JCR: journal citation report
OSRA: optical structure recognition application
QR: quick response
SDK: software development kits
VL: virtual laboratories
WoS: Web of Science

## References

[ref1] Rubilar M.; Chova L.; Martínez A.; Torres I. Designing of Augmented Reality Teaching-Learning Sequences to Promote the Accessibility and Vizualization of Complex Contents in Chemistry. In 11th International Conference on Education and New Learning Technologies; IATED, 2019; pp 8299–8303.

[ref2] Haghighatlari M.; Hachmann J. Advances of Machine Learning in Molecular Modeling and Simulation. Curr. Opin. Chem. Eng. 2019, 23, 51–57. 10.1016/j.coche.2019.02.009.

[ref3] Eriksen K.; Nielsen B. E.; Pittelkow M. Visualizing 3D Molecular Structures Using an Augmented Reality App. J. Chem. Educ. 2020, 97, 1487–1490. 10.1021/acs.jchemed.9b01033.

[ref4] Knierim P.; Kiss F.; Rauh M.; Schmidt A. Tangibility is Overrated: Comparing Learning Experiences of Physical Setups and their Virtual Equivalent in Augmented Reality. 19th International Conference on Mobile and Ubiquitous Multimedia; Association for Computing Machinery, 2020; pp 299–305.

[ref5] Brunnert R.; Bohrmann-Linde C.; Meuter N.; Pereira Vaz N.; Spinnen S.; Yurdanur Y.; Tausch M. W. The Fascinating World of Photochemistry. Video Tutorials for Core Concepts in Science Education. Educ. Quim. 2018, 29, 108–117. 10.22201/fq.18708404e.2018.1.63736.

[ref6] Abriata L. A. Building Blocks for Commodity Augmented Reality-based Molecular Visualization and Modelling in Web Browsers. PeerJ Comput. Sci. 2020, e260 10.7717/peerj-cs.260. PMC7924717 33816912

[ref7] Achuthan K.; Kolil V. K.; Diwakar S. Using Virtual Laboratories in Chemistry Classrooms as Interactive Tools Towards Modifying Alternate Conceptions in Molecular Symmetry. Educ. Inf. Technol. 2018, 23, 2499–2515. 10.1007/s10639-018-9727-1.

[ref8] Ratamero E. M.; Bellini D.; Dowson C. G.; Römer R. A. Touching Proteins with Virtual Bare Hands: Visualizing Protein-Drug Complexes and their Dynamics in Self-Made Virtual Reality Using Gaming Hardware. J. Comput.-Aided Mol. Des. 2018, 32, 703–709. 10.1007/s10822-018-0123-0. 29882064 PMC6028860

[ref9] Behmke D.; Kerven D.; Lutz R.; Paredes J.; Pennington R.; Brannock E.; Deiters M.; Rose J.; Stevens K. Augmented Reality Chemistry: Transforming 2-D Molecular Representations into Interactive 3-D Structures. Proceedings of the Interdisciplinary STEM Teaching and Learning Conference; Proceedings of the Interdisciplinary STEM Teaching and Learning Conference, 2018; Vol. 2, pp 5–11.

[ref10] Müller C.; Krone M.; Huber M.; Biener V.; Herr D.; Koch S.; Reina G.; Weiskopf D.; Ertl T. Interactive Molecular Graphics for Augmented Reality Using HoloLens. J. Integr. Bioinform. 2018, 15, 20180005 10.1515/jib-2018-0005. PMC6167047 29897886

[ref11] Liarokapis F.; Macan L.; Malone G.; Rebolledo-Mendez G.; De Freitas S. Multimodal Augmented Reality Tangible Gaming. Vis. Comput. 2009, 25, 1109–1120. 10.1007/s00371-009-0388-3.

[ref12] Damayanti L. A.; Ikhsan J. Augmented Chemistry Aldehida & Keton with 3 Dimensional (3D) Illustration. Supplement Book on Chemistry Learning. International Conference on Education, Mathematics and Science (ICEMS); AIP Publishing, 2017; Vol. 1847, p 050003.

[ref13] Astuti A. P.; Mawarsari V. D.; Purnomo H.; Sediyono E. The Use of Augmented Reality-Based Learning Media to Develop the Technology Literacy of Chemistry Teachers in the 21^st^ Century. In 3rd International Conference on Mathematics and Science Education; Habiddin H., Majid S., Suhadi I., Farida N., Dasna I., Eds.; AIP Publishing, 2020; Vol. 2215, p 020002.

[ref14] Nichele A.; do Canto L.; da Silva F. Augmented Reality: APPs for Teaching and Learning Chemistry. In 14th International Technology, Education and Development Conference; Chova L., Martinez A., Torres I., Eds.; INTED Proceedings, 2020; pp 7650–7655.

[ref15] Broman K.; Mårell-Olsson E.; Johnels D.; Andersson C. D.; Chorell E.; Westerlind U.; Bostrom J.; Norrby M. Spatial Ability in Organic Chemistry: Can Virtual and Augmented Reality be Valuable?. 7th Utvecklingskonferensen för Sveriges ingenjörsutbildningar; Luleå tekniska universitet, 2019.

[ref16] Tzima S.; Styliaras G.; Bassounas A. Augmented Reality Applications in Education: Teachers Point of View. Educ. Sci. 2019, 9, 99 10.3390/educsci9020099.

[ref17] Rosenberg L. B. The Use of Virtual Fixtures as Perceptual Overlays to Enhance Operator Performance in Remote Environments. Crew Systems Directorat Biodynamics and Biocommunications Division Wright-Patterson AFB OH45433-7901; Air Force Material Command: Ohio, 1992.

[ref18] Botden S. M. B. I.; Jakimowicz J. J. What is going on in Augmented Reality simulation in laparoscopic surgery?. Surg. Endosc. 2009, 23, 1693 10.1007/s00464-008-0144-1. 18813987 PMC2710490

[ref19] Yusof C. S.; Bai H.; Billinghurst M.; Sunar M. S. A review of 3D Gesture Interaction for Handheld Augmented Reality. J. Teknol. 2015, 78. 10.11113/jt.v78.6923.

[ref20] Palmarini R.; Erkoyuncu J. A.; Roy R.; Torabmostaedi H. A Systematic Review of Augmented Reality Applications in Maintenance. Robot Comput. Integr. Manuf. 2018, 49, 215–228. 10.1016/j.rcim.2017.06.002.

[ref21] Fombona J.; Pascual-Sevillano M.-d. -l. -Á.; González-Videgaray M. M-learning and Augmented Reality: A Review of the Scientific Literature on the WoS Repository. Comunicar 2017, 25, 63–72. 10.3916/c52-2017-06.

[ref22] Cheng K.-H.; Tsai C.-C. Affordances of Augmented Reality in Science Learning: Suggestions for Future Research. J. Sci. Educ. Technol. 2013, 22, 449–462. 10.1007/s10956-012-9405-9.

[ref23] Santos M. E. C.; Taketomi T.; Yamamoto G.; Rodrigo M. M. T.; Sandor C.; Kato H. Augmented Reality as Multimedia: The Case for Situated Vocabulary Learning. Res. Pract. Technol. Enhanc. Learn. 2016, 11, 1–23. 10.1186/s41039-016-0028-2. PMC6302860 30613237

[ref24] Dalim C. S. C.; Kolivand H.; Kadhim H.; Sunar M. S.; Billinghurst M. Factors Influencing the Acceptance of Augmented Reality in Education: A review of the Literature. J. Comput. Sci. 2017, 13, 581–589. 10.3844/jcssp.2017.581.589.

[ref25] Billinghurst M.; Kato H.; Poupyrev I. Tangible Augmented Reality. ACM Siggraph Asia 2008, 8, 1–10. 10.1145/1508044.1508051.

[ref26] Grunewald Nichele A.; do Nascimento G. Augmented Reality in Teaching Chemistry. 11th International Conference on Technology, Education and Development (INTED); INTED, 2017; pp 8736–8743.

[ref27] Fjeld M.; Voegtli B. M. Augmented Chemistry: an interactive educational workbench. Proceedings. International Symposium on Mixed and Augmented Reality; Institute of Electrical and Electronics Engineers, 2002; pp 259–321.

[ref28] Okamoto M.; Ishimura T.; Matsubara Y. AR-based Inorganic Chemistry Learning Support System Using Mobile HMD. 25th International Conference on Computers in Education (ICCE); Technology and Innovation - Computer-Based Educational Systems for the 21st Century, 2017; pp 511–513.

[ref29] Redó M. N.; Torres A. Q.; Quirós R.; Redó I. N.; Castelló J. B. C.; Camahort E. New Augmented Reality Applications: Inorganic Chemistry Education. Teaching through Multi-User Virtual Environments: Applying Dynamic Elements to the Modern Classroom; IGI Global, 2011.

[ref30] Faustino Andrade T.; Maier P.; Quintas M. R.; Klinker G.; Restivo M. T. Adding sensorial capabilities to the augmented chemical reactions application. 11th International Conference on Remote Engineering and Virtual Instrumentation; Institute of Electrical and Electronics Engineers, 2014; pp 217–218.

[ref31] Hou H.-T.; Lin Y.-C. The Development and Evaluation of an Educational Game Integrated with Augmented Reality and Virtual Laboratory for Chemistry Experiment Learning. 6th IIAI International Congress on Advanced Applied Informatics (IIAI-AAI); Institute of Electrical and Electronics Engineers, 2017; pp 1005–1006.

[ref32] Boletsis C.; McCallum S. The Table Mystery: An Augmented Reality Collaborative Game for Chemistry Education. In Serious Games Development and Applications. SGDA 2013. Lecture Notes in Computer Science; Ma M., Oliveira M. F., Petersen S., Hauge J. B., Eds.; Springer: Berlin, Heidelberg, 2013; p 86.

[ref33] Cai S.; Wang X.; Chiang F.-K. A case study of Augmented Reality simulation system application in a chemistry course. Comput. Hum. Behav. 2014, 37, 31–40. 10.1016/j.chb.2014.04.018.

[ref34] Chen Y.-C. A study of comparing the use of augmented reality and physical models in Chemistry education. In VRCIA 06 Proceedings of the 2006 ACM international conference on Virtual reality continuum and its applications; Association for Computing Machinery, 2006; pp 369–372.

[ref35] Yaman O.; Karakose M. Development of Image Processing Based Methods Using Augmented Reality in Higher Education. 15th International Conference on Information Technology Based Higher Education and Training (ITHET); Institute of Electrical and Electronics Engineers, 2016; pp 1–5.

[ref36] Martínez-Hung H.; García-López A.; Escalona-Arranz J. C. Modelos de Realidad Aumentada aplicados a la enseñanza de la Química en el nivel universitario. Rev. cuba. quím. 2017, 29, 13–25.

[ref37] Ruiz-Cerrillo S. Augmented Reality and Learning in Organic Chemistry. Apertura 2020, 12, 106–117. 10.32870/ap.vl2nl.1853.

[ref38] Vega J. C.; Vega Garzón M. L.; Galembeck E. Using Augmented Reality to Teach and Learn Biochemistry. Biochem. Mol. Biol. Educ. 2017, 45, 417–420. 10.1002/bmb.21063. 28436090

[ref39] Williams A. J.; Ekins S.; Clark A. M.; Jack J. J.; Apodaca R. L. Mobile apps for chemistry in the world of drug discovery. Drug Discov. Today 2011, 16, 928–939. 10.1016/j.drudis.2011.09.002. 21924376

[ref40] Alavi S. Molecular Simulations: Fundamentals and Practice; Wiley-VCH Verlag GmbH, 2020.

[ref41] Romeo A.; Iacovelli F.; Falconi M. Targeting the SARS-CoV-2 Spike Glycoprotein Prefusion Conformation: Virtual Screening and Molecular Dynamics Simulations Applied to the Identification of Potential Fusion Inhibitors. Virus Res. 2020, 286, 198068 10.1016/j.virusres.2020.198068. 32565126 PMC7301794

[ref42] Fatemah A.; Rasool S.; Habib U. Interactive 3D Visualization of Chemical Structure Diagrams Embedded in Text to Aid Spatial Learning Process of Students. J. Chem. Educ. 2020, 97, 992–1000. 10.1021/acs.jchemed.9b00690.

[ref43] Coster A. MoleculAR: an augmented reality app for organic chemistry. https://organicchemexplained.com/molecular-augmented-reality-app (accessed August, 2021).

[ref44] Grunewald A.; Zielinski L.; Nunes F. Augmented Reality: Apps for Teaching and Learning Chemistry. 14th International Technology, Education and Development Conference; Chova L., Martinez A., Torres I., Eds.; IATED, 2020; pp 7650–7655.

[ref45] Swamy K L N.; Chavan P. S.; Murthy S. StereoChem: Augmented Reality 3D Molecular Model Visualization App for Teaching and Learning Stereochemistry. 8th IEEE International Conference on Advanced Learning Technologies (ICALT); Institute of Electrical and Electronics Engineers, 2018; pp 252–256.

[ref46] Ishimura T.; Okamoto M.; Matsubara Y. Smartphone-Based Inorganic Chemistry Learning Support System Using AR Approach. 13th International Conference on Industrial Management (ICIM); International Conference on Industrial Management, 2016; pp 502–508.

[ref47] Aw J. K.; Boellaard K. C.; Tan T. K.; Yap J.; Loh Y. P.; Colasson B.; Blanc É.; Lam Y.; Fung F. M. Interacting with Three-Dimensional Molecular Structures Using an Augmented Reality Mobile App. J. Chem. Educ. 2020, 97, 3877–3881. 10.1021/acs.jchemed.0c00387.

[ref48] Nunes De Vasconcelos G.; Malard M.; Van Stralen M.; Campomori M.; Canavezzi De Abreu S.; Lobosco T.; Gomes I.; Duarte L.; Lima C. Do we still need CAVEs?. Architecture in the Age of the 4th Industrial Revolution - Proceedings of the 37th Ecaade and 23rd SIGraDi Conference; Sousa J. P., Xavier J. P., Castro Henriques G., Eds.; University of Porto: Porto, Portugal, 2019; pp 133–142.

[ref49] Goddard T. D.; Brilliant A. A.; Skillman T. L.; Vergenz S.; Tyrwhitt-Drake J.; Meng E. C.; Ferrin T. E. Molecular Visualization on the Holodeck. J. Mol. Biol. 2018, 430, 3982–3996. 10.1016/j.jmb.2018.06.040. 29964044 PMC6223615

[ref50] Grandi J. G.; Debarba H. G.; Bemdt I.; Nedel L.; Maciel A. Design and Assessment of a Collaborative 3D Interaction Technique for Handheld Augmented Reality. IEEE Conference on Virtual Reality and 3D User Interfaces (VR); Institute of Electrical and Electronics Engineers, 2018; pp 49–56.

[ref51] Qian J.; Ma J.; Li X.; Attal B.; Lai H.; Tompkin J.; Hughes J. F.; Huang J. Portal-ble: Intuitive Free-Hand Manipulation in Unbounded Smartphone-Based Augmented Reality. 32nd Annual ACM Symposium on User Interface Software and Technology; Association for Computing Machinery, 2019; pp 133–145.

[ref52] Bach B.; Sicat R.; Beyer J.; Cordeil M.; Pfister H. The Hologram in my Hand: How Effective is interactive Exploration of 3D Visualizations in Immersive Tangible Augmented Reality?. IEEE Trans. Vis. Comput. Graph. 2018, 24, 457–467. 10.1109/TVCG.2017.2745941. 28866590

[ref53] Lam M.; Tee H.; Nizam S.; Hashim N.; Suwadi N.; Tan S.; Abd Majid N.; Arshad H.; Liew S. Interactive Augmented Reality with Natural Action for Chemistry Experiment Learning. TEM J. 2020, 9, 351–360. 10.18421/TEM91-48.

[ref54] Tee N. Y. K.; Gan H. S.; Li J.; Cheong B. H.-P.; Tan H. Y.; Liew O. W.; Ng T. W. Developing and Demonstrating an Augmented Reality Colorimetric Titration Tool. J. Chem. Educ. 2018, 95, 393–399. 10.1021/acs.jchemed.7b00618.

[ref55] Wan A. T.; San L. Y.; Omar M. S. Augmented Reality Technology for Year 10 Chemistry Class: Can the Students Learn Better?. Int. J. Comput. Assist. 2018, 8, 45–64. 10.4018/IJCALLT.2018100104.

[ref56] Abdinejad M.; Talaie B.; Qorbani H. S.; Dalili S. Student Perceptions Using Augmented Reality and 3D Visualization Technologies in Chemistry Education. J. Sci. Educ. Technol. 2021, 30, 87–96. 10.1007/s10956-020-09880-2.

[ref57] Nechypurenko P. P.; Starova T. V.; Selivanova T. V.; Tomilina A. O.; Uchitel A. D. Use of Augmented Reality in Chemistry Education. In 1st International Workshop on Augmented Reality in Education (AREdu 2018); Kiv A. E., Soloviev V. N., Eds.; Kryvyi Rih State University: Kryvyi Rih, Ukraine, 2018. CEUR 2257, 15–23. http://ceur-ws.org/Vol-2257/paper02.pdf (accessed August 2021).

[ref58] Da Silva M. M.; Teixeira J. M. X.; Cavalcante P. S.; Teichrieb V. Perspectives on How to Evaluate Augmented Reality Technology Tools for Education: A Systematic Review. J. Brazilian Comput. Soc. 2019, 25, 1–18. 10.1186/s13173-019-0084-8.

[ref59] Chen S.-Y.; Liu S.-Y. Using Augmented Reality to Experiment with Elements in a Chemistry Course. Comput. Hum. Behav. 2020, 111, 106418 10.1016/j.chb.2020.106418.

[ref60] Ozdemir M.; Sahin C.; Arcagok S.; Demir M. K. The effect of Augmented Reality Applications in the Learning Process: A Meta-Analysis Study. Eurasian J. Educ. Res. 2018, 18, 165–186. 10.14689/ejer.2018.74.9.

[ref61] Garcia Fracaro S.; Chan P.; Gallagher T.; Tehreem Y.; Toyoda R.; Bernaerts K.; Glassey J.; Pfeiffer T.; Slof B.; Wachsmuth S.; Wilk M. Towards Design Guidelines for Virtual Reality Training for the Chemical Industry. Educ. Chem. Eng. 2021, 36, 12–23. 10.1016/j.ece.2021.01.014.

[ref62] Wolle P.; Müller M. P.; Rauh D. Augmented Reality in Scientific Publications-Taking the Visualization of 3D Structures to the Next Level. ACS Chem. Biol. 2018, 13, 496–499. 10.1021/acschembio.8b00153. 29544257

[ref63] Matthews D. Virtual-Reality Applications Give Science a New Dimension. Nature 2018, 557, 127–128. 10.1038/d41586-018-04997-2. 29713071

[ref64] Sanii B. Creating Augmented Reality USDZ Files to Visualize 3D Objects on Student Phones in the Classroom. J. Chem. Educ. 2020, 97, 253–257. 10.1021/acs.jchemed.9b00577.

[ref65] Sliwinski E.; Kabeshov M.; Ley S. HTMoL – AR plugin. https://github.com/es605/HTMoLAR (accessed May, 2019).

[ref66] Irwansyah F. S.; Yusuf Y. M.; Farida I.; Ramdhani M. A. Augmented Reality (AR) Technology on the Android Operating System in Chemistry Learning. 2nd AASEC 2017; Abdullah A., Nandiyanto A., Widiaty I., Eds.; IOP Publishing, 2018; Vol. 288, p 012068.

[ref67] Jiménez Z. A. Teaching and Learning Chemistry Via Augmented and Immersive Virtual Reality. Technology Integration in Chemistry Education and Research (TICER); American Chemical Society: Washington, 2019; Vol. 3, pp 31–52.

[ref68] Roqueta M. L. Augmented Reality (AR) in teaching Chemistry. In Virtual Congress; Pons A., Mir S., Benavent J., Lopez I., Pastor C., Huerta P., Peris A., Eds.; ATIDES: Garcia, 2018; p 19.

[ref69] Hung H.; Lopez A.; Gonzalez O.; Verdecias I. Augmented Reality on the Coordination Chemistry and Solid Structure Teaching. Atenas 2019, 46, 111–125.

[ref70] Eljack S.; Alfayez F.; Suleman N. Organic Chemistry Virtual Laboratory Enhancement. Int. J. Appl. Math. Comput. Sci. 2020, 15, 309–323.

[ref71] Agbonifo O. C.; Sarumi O. A.; Akinola Y. M. A Chemistry Laboratory Platform Enhanced with Virtual Reality for Students’ Adaptive Learning. Res. Learn. Technol. 2020, 28, 2419 10.25304/rlt.v28.2419.

[ref72] Quintero J.; Baldiris S.; Rubira R.; Cerón J.; Velez G. Augmented Reality in educational inclusion. A systematic review on the last decade. Front. Psychol. 2019, 10, 1835 10.3389/fpsyg.2019.01835. 31456716 PMC6700208

[ref73] Schmid J. R.; Ernst M. J.; Thiele G. Structural Chemistry 2.0: Combining Augmented Reality and 3D Online Models. J. Chem. Educ. 2020, 97, 4515–4519. 10.1021/acs.jchemed.0c00823.

[ref74] Barroso Osuna J.; Gutiérrez-Castillo J. J.; Llorente-Cejudo M. d. C.; Valencia Ortiz R. Difficulties for the incorporation of Reality Augmented in university teaching: visions from the experts. NAER 2019, 8, 126–141. 10.7821/naer.2019.7.409.

[ref75] Aristov M. M.; Moore J. W.; Berry J. F. Library of 3D Visual Teaching Tools for the Chemistry Classroom Accessible via Sketchfab and Viewable in Augmented Reality. J. Chem. Educ. 2021, 98, 3032–3037. 10.1021/acs.jchemed.1c00460.

[ref76] Kelly R.; Akaygun S. Visualizations and Representations in Chemistry Education. Chem. Educ. Res. Pract. 2019, 20, 657–658. 10.1039/C9RP90009H.

[ref77] Hanson-Heine M. W. D.; Ashmore A. P. Computational Chemistry Experiments Performed Directly on a Blockchain Virtual Computer. Chem 2020, 11, 4644–4647. 10.1039/d0sc01523g. PMC8159212 34122919

[ref78] Juárez-Jiménez J.; Tew P.; O′Connor M.; Llabrés S.; Sage R.; Glowacki D.; Michel J. Combining Virtual Reality Visualization with Ensemble Molecular Dynamics to Study Complex Protein Conformational Changes. J. Chem. Inf. Model. 2020, 60, 6344–6354. 10.1021/acs.jcim.0c00221. 33180485

[ref79] Sakshuwong S.; Weir H.; Raucci U.; Martinez T. Bringing Chemical Structures to Life with Augmented Reality, Machine Learning and Quantum Chemistry. MolAR: Bringing Chemical Structures to Life with Augmented Reality and Machine Learning; ChemRxiv: Cambridge Open Engage: Cambridge, 2021.

[ref80] Artrith N.; Butler K. T.; Coudert F.-X.; Han S.; Isayev O.; Jain A.; Walsh A. Best practices in machine learning for chemistry. Nat. Chem. 2021, 13, 505–508. 10.1038/s41557-021-00716-z. 34059804

