# Supplementary material for: Augmented Reality, a Review of a Way to Represent and Manipulate 3D Chemical Structures
Source: J Chem Inf Model. 2022 Apr 4;62(8):1863–72. doi: 10.1021/acs.jcim.1c01255 (PMC9044447; doi:10.1021/acs.jcim.1c01255)
Supplement: Supplementary file 1 — ci1c01255_si_001.pdf [file ci1c01255_si_001.pdf]

Supporting Information

Augmented Reality, a review of a way to represent and manipulate 3D chemical structures

Alba Fombona-Pascual[(1)], Javier Fombona[(2)] and Ruben Vicente[(3)]

[(1)] Institute IMDEA Energy - Móstoles Technology, Avda. Ramón de la Sagra, 3, 28935 Madrid, Spain.

[(2)] Education Sciences Department, University of Oviedo, Aniceto Sela, 33005 Oviedo, Spain.

[(3)] Organic Chemistry Department, University of Oviedo, Julian Clavería, 33005 Oviedo, Spain.

Corresponding Author: Javier Fombona fombona@uniovi.es

Phone: +34 9851032885

Data and Software Availability

All data used are accessible and all methods are described. In this sense, the procedure and method used in this research can be reproduced following the phases described in this manuscript (Phase 1: Selection of the sample of documents, and Phase 2: Content analysis of the documents). In this sense the methodology is rigorous, and the data are accessible from a public source, such as the Web of Science document base. Both the documents and the software described have been tested by third parties, and these experiences have their corresponding citations and bibliographic references.

The software mentioned in the article is available for evaluation and replication of assays:

- PubChem, Software on line for PC to show a 3D simulation of the Caffeine molecule (1,3,7-Trimethylxanthine), (Figure 1). Software accessibility: https://pubchem.ncbi.nlm.nih.gov/

- Molecule3D 3.9, 3D simulation software APP off line for smartphone to show the Caffeine molecule (1,3,7-Trimethylxanthine), (Figure 1). Software accessibility: https://play.google.com/store/apps/details?id=com.molviewfree&hl=en_US&gl=US

- Model AR1.3, Augmented Reality software for visualization of the molecule Caffeine (1,3,7-Trimethylxanthine), (Figure 2). Software accessibility: https://play.google.com/store/apps/details?id=com.alchemie.modelset&hl=en_US&gl=US

- QRChem, Software for PC on line to show Augmented Reality structures through a QR code of the molecule Caffeine (1,3,7-Trimethylxanthine), (Figure 2). Software accessibility: https://qrchem.net

- Nanome Virtual Reality, Software to show a 3D molecule Caffeine (1,3,7-Trimethylxanthine) (Figure 3). Software accessibility: https://nanome.ai/

- SponholtzProductions, Augmented Reality software on line, to show the Methylamine molecule, (Figure 4). Software accessibility: https://sponholtzproductions.com/bin-release/ar.html

- Web of Science Clarivate Analytics (WoS) is available from FECYT Spain, Website used to access the different researchers analyzed https://www.recursoscientificos.fecyt.es/

- BIbExcel 2016.2.20, software to analyze texts, Software accessibility: https://homepage.univie.ac.at/juan.gorraiz/bibexcel/bibexcel.exe

- Aquad7, software to analyze texts. Software accessibility: http://www.aquad.de

Other software tested by third parties and only mentioned in this article: AltPDB, ARChemEx, ARchemy, ARKimia Kit, Augmented Chemistry, Augmented Reality Metabolic Pathways ARMET, CCP4MG, ChemPreview, Chimera, Coot, Elements 4D, Isolde, Iview, Jmol, Molecular Zoo, NumPy, Optical Structure Recognition Application (OSRA), PyMOL, Rasmol, UCSF, Unity3d, VMD, Vuforia, and the list in Table 2.

Table S1. Original list with 315 applications for visualization that we have analyzed to select 175 applications.

- 3DB Unity
- 3dmol.js
- Abalone
- ABINIT
- AbM
- ACD/ChemSketch
- ACES
- ADF
- Advanced Simulation Library
- Agile Molecule
- AIMAll
- AltPDB Protein-viewer-activity
- AMBER
- Amira
- AMPAC
- AMSOL
- Amsterdam Density Functional
- APEX-3D
- Aqion
- Ascalaph Designer
- Atomistix
- Autochem
- AutoDock
- Avizo
- Avogadro
- Babel
- BALL
- BigDFT
- BioBlender
- Biskit
- BKChem
- Bondit
- BRAGI
- CAChe
- CADPAC
- Cambridge Structural Database (CSD)
- Cantera
- Car–Parrinello molecular dynamics
- CASINO
- CASTEP
- CAVEAT
- CCDVault
- CCP4MG
- CHARMM
- Chem 4-d
- Chemcraft
- ChemDBS-3D
- ChemDraw
- Chemical shift index
- Chemical WorkBench
- Chemicalize
- Chemistry Development Kit
- Chemitorium
- CHEMKIN
- ChemOffice
- Chemsketch
- ChemSpider (JS-mol)-Web
- ChemVLab+ WEB
- ChemWindow
- Chem-X
- CHIME
- Chimera
- CLIP
- ClogP
- CMR
- Cn3D
- COLUMBUS
- Comparison of nucleic acid simulation
- Composer
- Computational Center Macromolecular S.
- CONCORD
- CONQUEST
- COSILAB
- Coot Crystallographic Object-Oriented T.
- CP2K
- Crystal
- CrystalMaker
- CS ChemOffice Pro
- CS23D
- Cuemol
- Dalton
- DelPhi
- DGEOM 95
- DGII
- Dirac
- DISCO
- Discover
- Discovery Studio
- DMol
- DOCK
- DP code
- Drug Bank WEB
- DSSP
- EGO
- enCIFer
- ePMV
- Eulim
- EXC code
- Extensible Computational Chemistry E.
- EzMol Web
- FDMNES
- FHI-aims
- Firefly
- Foldit
- FreeON
- Gabedit
- GALAXY
- GAMESS
- GASP
- Gaussian
- GausView
- GEMM
- GeNMR
- GERM
- Ghemical-GMS
- GLARE
- Glauber
- GNU Octave
- gOpenMol
- GPCR-ModSim
- Graffiti
- GRAMM
- GRASP
- GROMACS
- GROMOS
- HASL
- hBar Lab
- HBPLUS
- Hingefind Algorithm
- HINT
- HOLLOW
- HONDO
- HORTON
- HyperChem
- Hypercube
- ICM Chemist
- ICM-Browser
- IcmJS
- Iditis
- IMol
- Insight II
- Internal Coordinate Mechanics
- ISIS
- IsisDraw
- Iview WEBGL
- Jaguar
- JChemPaint
- JME Molecule Editor
- Jmol JavaScript-Based Molecular JSmol
- JOELib
- Joint Expert Speciation System
- Jolecule Protein viewer
- Kekulé Program
- Khimera
- Kinemage, MAGE & King
- Kinetic PreProcessor
- Leapfrog
- LigPlot
- LiSiCA
- List of computer-assisted organic synth.
- List of protein-ligand docking software
- Look/GeneMine
- Loopy
- Ludi
- Luscus
- MacroModel
- MADNESS
- Marvin,
- Match
- Materials Studio
- MC-SYM
- MDL Chime
- MembraneEditor CELLmicrocosmos
- Mercury Crystal Structure Visualization
- MGLTools (Python & AutoDock)
- Midas+
- MidasPlus
- MM3
- MODELLER
- Moil
- Mol2Mol
- MOLCAS
- Molconn-z
- Molden UNIX
- Moldraw
- Molecular design software
- Molecular modeling on GPUs
- Molecular Operating Environment
- Molecular Workbench
- Molecular mechanics modeling
- Molecular Rift
- Molecule
- Molegro Virtual Docker
- MOLEKEL
- MOLGEN+
- MolPOV
- MolPro
- MolScript
- Molsketch
- MoluCAD
- MolView
- MOPAC
- MOPLOT
- MovieMol
- MPQC
- NAMD
- NAMOT
- Nanome PC
- NanoVision
- NAOMI
- NEST
- Newton-X
- NGL viewer molecules
- NT-NMRPipe
- NWChem
- OctaDist
- Octopus
- OELib
- ONETEP
- Open Babel
- OpenAtom
- Optical Structure Recognition
- ORCA (Quantum Chemistry Program)
- PARSEC
- PDB Protein Data Bank
- Perse Visualizer
- Pipeline Pilot
- PKalc
- PLATO
- POLYVIEW
- PovChem
- PQS Parallel Quantum
- ProBiS333
- Profix
- PrologD
- PrologP
- PROSA II
- Prosat+
- Protein chemical shift
- Protein Data Bank (PDB)
- Protein Explorer
- PSI
- Psi88 Fortran
- PubChem Web
- PV-JavaScript Protein Viewer
- PyMol
- PyQuante
- PySCF
- Python Molecular Viewer PMV
- Qbox
- Q-Chem
- QUANTA
- Quantemol-DB
- Quantum chemistry solid-state
- Quantum ESPRESSO
- QUEST
- QuteMol
- Random coil index
- RasMol
- Raster3D
- RasTop
- RCSB MBT Viewers
- RefDB
- Ribbons
- RINalyzer
- RMG
- RmscopII
- RPluto
- Rubychem
- SAMSON
- SCAP
- SCARECROW
- Schrödinger
- Scigress
- Scilab
- SCULPT
- Setor
- SHIFTCOR
- ShelXle
- SIESTA
- Spartan
- Spatial Discrete Event Simulat.
- STRUCTURELAB
- Swiss PDB viewer
- SYBYL
- Sybyl-X
- TeraChem
- Tessel Fortran
- Tinker Molecular Modeling
- Turbomole
- UCSF
- Ugene
- UHBD
- UniChem
- Valence bond programs
- VASPMO
- Vega
- VIDA
- Vienna Ab initio Simulation
- ViewMol
- Vibeplot
- VisualMolecularDynamicVMD
- Virtual Chemistry 3D
- WebLab
- What If
- WIEN2k
- XDrawChem
- XMol
- X-PLOR
- XtalView
- XyMTeX
- YAMBO code
- Yasara
- Zeus
- ZINDO

Table S2. Analysis and brief description of the software for on-screen visualization and manipulation.

Name	Link	Description
3DB Unity	https://assetstore.unity.com/packages/3d/props/industrial/chemistry-bundle-16668#content	Virtualware: pack of chemistry related objects composed of 38 items. Models are low-mid poly models with diffuse textures up to 2048 pixels. Not interesting here.
3dmol.js	https://3dmol.csb.pitt.edu/	An object-oriented JavaScript library for visualizing molecular data.
Abalone	http://www.biomolecular-modeling.com/Abalone/index.html	Molecular simulations of proteins, hydrocarbons, perfluorocarbons, heavy elements, any molecule via default FF.
ABINIT	https://www.abinit.org/	ABINIT is a software suite to calculate the optical, mechanical, vibrational, and other observable properties of materials. Starting from the quantum equations of density functional theory, you can build up to advanced applications with perturbation theories based on DFT, and many-body Green's functions (GW and DMFT). ABINIT can calculate molecules, nanostructures and solids with any chemical composition, and comes with several complete and robust tables of atomic potentials.
ACD/ChemSketch	https://www.acdlabs.com/products/draw_nom/draw/chemsketch/?gclid=CjwKCAjww-CGBhALEiwAQzWxOjGHcOKf-Z_NhWzd4gggFH6Knkq_4GxAUoCLsw6Go03c9cOrMRGq3BoCc04QAvD_BwE	ACD/ChemSketch is an easy-to-use, chemically intelligent molecular structure drawing application, with more than 2 million users worldwide. Draw chemical structures, reactions, and schema, and access a variety of graphical tools and templates.Generate names from molecular structure. Calculate molecular properties from chemical structure. Create professional reports, presentations, and publication-ready figures. Communicate scientific information with clarity and ease.
ACES II	http://slater.chemie.uni-mainz.de/xaces2/index.php/Main/HomePage	ACES II (Advanced Concepts in Electronic Structure) is a series of programs for performing high-level quantum chemical ab initio calculations. Its major strength is the accurate calculation of atomic and molecular energies as well as properties using "many-body" techniques such as many-body perturbation theory (MBPT) and, in particular coupled-cluster techniques to treat electron correlation. ACES II offers a number of unique features especially for the accurate calculation of molecular properties.
ADF	https://www.scm.com/product/adf/	Amsterdam Density Functional (ADF) is particularly strong in understanding and predicting structure, reactivity (catalysis), and spectra of molecules. Density Functional Theory (DFT) calculations are easily prepared and analyzed with our integrated graphical user interface. ADF is frequently used for studying transition metal complexes and molecules with heavy atoms, since all elements in the periodic table can be modeled accurately and efficiently with the ZORA relativistic approach and Slater Type orbital (STO) all-electron basis sets. With these features, ADF offers unique capabilities to predict molecular properties of nanoparticles and organic electronics materials.
Advanced Simulation Library (ASL)	http://asl.org.il/	Advanced Simulation Library (ASL) is a free and open source hardware accelerated multiphysics simulation platform (and an extensible general purpose tool for solving Partial Differential Equations). Its computational engine is written in OpenCL and utilizes matrix-free solution techniques which enable extraordinarily high performance, memory efficiency and deployability on a variety of massively parallel architectures, ranging from inexpensive FPGAs, DSPs and GPUs up to heterogeneous clusters and supercomputers. The engine is hidden entirely behind simple C++ classes, so that no OpenCL knowledge is required from application programmers. Mesh-free, immersed boundary approach allows one to move from CAD directly to simulation drastically reducing pre-processing efforts and amount of potential errors. ASL can be used to model various coupled physical and chemical phenomena and employed in a multitude of fields: computational fluid dynamics, virtual sensing, industrial process data validation and reconciliation, image-guided surgery, computer-aided engineering, design space exploration, crystallography, etc.
AIMAll	http://aim.tkgristmill.com/	AIMAll is a quantum chemistry software package for performing comprehensive, quantitative and visual QTAIM analyses of molecular systems - starting from molecular wavefunction data.
AltPDB Protein-viewer-activity	https://github.com/AltPDB	AltSpaceVR application for viewing and discussing PDB protein structure.
AMBER	https://ambermd.org/	Amber is a suite of biomolecular simulation programs. it is a set of molecular mechanical force fields for the simulation of biomolecules (these force fields are in the public domain, and are used in a variety of simulation programs). Second, it is a package of molecular simulation programs which includes source code and demos.
Amira	https://www.thermofisher.com/es/es/home/industrial/electron-microscopy/electron-microscopy-instruments-workflow-solutions/3d-visualization-analysis-software/amira-advanced-image-processing-quantification.html	The XImagePAQ extension for Thermo Scientific Amira Software provides a variety of image processing and quantification tools. These tools enable the user to perform image enhancement operations, create simplified and automated segmentation workflows, and perform extensive measurement and quantification tasks on objects in the image such as cells, bones, and other tissue features.
AMPAC	http://www.semichem.com/	AMPAC™ is Semichem's flagship product and is a complete semiempirical quantum mechanical program. AMPAC™ also includes a graphical user interface (GUI) for building and visualization.
AMSOL	https://license.umn.edu/product/amsol-71-software-to-calculate-free-energies-of-solvation	AMSOL is a computational chemistry software program that calculates the free energy of a solvated molecule. The software application calculates the change in energy when molecules are dissolved in water or an organic solvent. The results may also be used to calculate partition coefficients and their logarithms (Log P).

Amsterdam Density Functional	https://www.scm.com/product/ams/	Amsterdam Density Functional (ADF) is a program for first-principles electronic structure calculations that makes use of density functional theory (DFT). AMS driver is a central tool for complex potential energy tasks, such as molecular dynamics, Monte Carlo, PES scans, and finding transition states. The AMS driver can be used with the modules in the Amsterdam Modeling Suite; ADF, BAND, DFTB, MOPAC, ReaxFF, and UFF. Not interesting here.
Ansys Chemkin-Pro	https://www.ansys.com/products/fluids/ansys-chemkin-pro	Ansys Chemkin-Pro is the industry leader for modeling complex, chemically reacting systems.
Aqion	https://www.aqion.de/	Free hydrochemistry and water analysis software. Not interesting here.
Ascalaph Designer	http://www.biomolecular-modeling.com/Ascalaph/Ascalaph_Designer.html	Ascalaph Designer is a general-purpose program for molecular dynamic simulations. Under a single graphical environment are represented as their own implementation of molecular dynamics as well as the methods of classical and quantum mechanics of popular programs.
Atomistic	https://www.scientec.com.mx/atomistic	Atomistic ToolKit ATK Electronic. ATK offers capabilities to simulate electrical transport properties of nanodevices at the atomic scale. Based on an open architecture that integrates a scripting language with a graphical user interface, ATK is a platform for studies in nanoelectronics, using both Exact First Principles (DFT) and classical fast and potential semi-empirical methods. ATK includes a electrostatic model to enable realistic simulations of nanoscale transistor structures.
Autochem	https://www.techbriefs.com/component/content/article/tb/pub/techbriefs/software/458	AutoChem is a suite of Fortran 90 computer programs for the modeling of kinetic reaction systems. AutoChem performs automatic code generation, symbolic differentiation, analysis, and documentation. It produces a documented stand-alone system for the modeling and assimilation of atmospheric chemistry.
AutoDock	http://autodock.scripps.edu/	AutoDock is a suite of automated docking tools. It is designed to predict how small molecules, such as substrates or drug candidates, bind to a receptor of known 3D structure.
Avizo	https://www.thermofisher.com/es/es/home/industrial/electron-microscopy/electron-microscopy-instruments-workflow-solutions/3d-visualization-analysis-software/avizo-materials-science.html	Materials characterization and quality control. 3D visualization and analysis of your imaging data allows you to better understand your materials structure, properties and performances. Avizo Software provides optimized workflows for advanced materials characterization and quality control.
Avogadro	https://avogadro.cc/	Avogadro is an advanced molecule editor and visualizer designed for cross-platform use in computational chemistry, molecular modeling, bioinformatics, materials science, and related areas. It offers flexible high quality rendering and a plugin architecture.
Babel	https://sourceforge.net/projects/openbabel/	Open Babel is a chemical toolbox designed to speak the many languages of chemical data. It's an open, collaborative project allowing anyone to search, convert, analyze, or store data from molecular modeling, chemistry, biochemistry, or related areas.
BALL	https://ball-project.org/	Rapid Software Prototyping can significantly reduce development times in the field of Computational Molecular Biology and Molecular Modeling. BALL (Biochemical ALgorithms Library) is an application framework implemented in C++ that has been specifically designed for this purpose. It provides an extensive set of data structures as well as classes for Molecular Mechanics, solvation methods, comparison and analysis of protein structures, file import/export, and visualization.
BigDFT	https://bigdft.org/	The linear scaling approach of BigDFT enables DFT simulations for systems containing thousands of atoms.
BioBlender	http://www.bioblender.org/	BioBlender is a software package built on the open-source 3D modeling software Blender. Biology works at nanoscale, with objects invisible to the human eye. With BioBlender it is possible to show some of the characters that populate our cells, based on scientific data and the 3D manipulation.
BIOVIA	https://www.3ds.com/products-services/biovia/disciplines/molecular-modeling-and-simulation/	Drug Design, QM, MM. Using Materials Studio, researchers in many industries are engineering better performing materials of all types, including catalysts, polymers, composites, metals, alloys, pharmaceuticals, batteries and more.
Biskit	http://biskit.pasteur.fr/	Biskit is a modular, object-oriented Python library for structural bioinformatics research. It facilitates the manipulation and analysis of macromolecular structures, protein complexes, and molecular dynamics trajectories. For efficient number crunching, Biskit objects tightly integrate with numpy (Numeric Python). Biskit also offers a platform for the integration of external programs and new algorithms into complex workflows.
BKChem	https://bkchem.zirael.org/	BKChem is a free chemical drawing program. BKChem is written in Python, an interpreted and programming language.
BRAGI	https://bragi.helmholtz-hzi.de/downloads.html	BRAGI is a well-established package for viewing and modeling of three-dimensional (3D) structures of biological macromolecules. BRAGI enables you to view and explore the 3D structure of any macromolecule. It can explore proteins, DNA, RNA, carbohydrates, and complexes, such as between transcriptional regulatory proteins and DNA, or enzymes and drugs.
CADPAC	https://materialsdata.nist.gov/handle/11256/453?show=full	CADPAC, the Cambridge Analytic Derivatives Package, is a suite of programs for ab initio computational chemistry calculations. It is capable of molecular Hartree–Fock calculations, Møller–Plesset calculations, various other correlated calculations and Density Functional Theory calculations.

Cambridge Structural Database (CSD)	https://library.bath.ac.uk/chemistry-software/ccdc	The Cambridge Structural Database (CSD) contains over 900,000 small-molecule organic and metal-organic crystal structures. It is provided by the Cambridge Crystallographic Data Centre (CCDC).
Cantera	https://cantera.org/	Cantera automates the chemical kinetic, thermodynamic, and transport calculations so that the users can incorporate detailed chemical thermo-kinetics and transport models into their calculations.
Car–Parrinello molecular dynamics	https://bioexcel.eu/software/cpmd/	The CPMD code is a parallelized plane wave/pseudopotential implementation of Density Functional Theory, particularly designed for ab-initio molecular dynamics. CPMD is currently the most HPC code that allows performing quantum molecular dynamics simulations by using the Car-Parrinello molecular dynamics scheme. CPMD simulations are usually restricted to systems of few hundred atoms. In order to extend its domain of applicability to larger biologically relevant systems, a hybrid quantum mechanical/molecular mechanics (QM/MM) interface, employing routines from the GROMOS96 molecular dynamics code, has been developed.
CASINO	https://vallico.net/casinoqmc/what-is-casino/	CASINO is a computer program system for performing quantum Monte Carlo (QMC) electronic structure calculations that has been developed by a group of researchers initially working in the Theory of Condensed Matter group in the Cambridge University physics department, and their collaborators, over more than 20 years. It is capable of calculating accurate solutions to the Schrödinger equation of quantum mechanics for realistic systems built from atoms.
CASTEP	http://www.castep.org/	CASTEP is a leading code for calculating the properties of materials from first principles. Using density functional theory, it can simulate a wide range of properties of materials proprieties including energetics, structure at the atomic level, vibrational properties, electronic response properties etc. In particular it has a wide range of spectroscopic features that link directly to experiment, such as infra-red and Raman spectroscopies, NMR, and core level spectra.
CAVEAT	http://www.cchem.berkeley.edu/pabgrp/Data/caveat.html	The program CAVEAT is a program to facilitate the design of organic molecules. Key innovations introduced in CAVEAT are a focus on relationships between bonds and the provision of automated methods to identify and classify structural frameworks.
CCDVault	https://www.collaborativedrug.com/	CDD Vault allows organize chemical structures and biological study data, and collaborate work with internal or external partners through a web interface.
CCP4MG	http://legacy.ccp4.ac.uk/	CCP4mg is a molecular-graphics program that is designed to give access to both straightforward and complex static and dynamic representations of macromolecular structures.
CHARMM	https://www.charmm.org/	Chemistry at HARvard Macromolecular Mechanics. A molecular simulation program with broad application to many-particle systems with a comprehensive set of energy functions, a variety of enhanced sampling methods, and support for multi-scale techniques including QM/MM, MM/CG, and a range of implicit solvent models
Chem 4-d	http://www.cheminnovation.com/products/chem4d.asp	Chem4D Graph module that creates multi-line graphs of different styles. It supports non-linear and linear curve fitting, response curve fitting and data analysis. The program allows you to create structures simply by entering molecular names. It assigns systematic names to structures. It includes a full set of tools for drawing, text and structure editing, and labeling.
Chemcraft	https://www.chemcraftprog.com/	Chemcraft is a graphical program for working with quantum chemistry computations. It is a convenient tool for visualizing computed results and preparing new jobs for a calculation. Chemcraft is mainly developed as a graphical user interface for the GAMESS (US version and the PCGamess/Firefly) and Gaussian program packages. For dealing with other computation types, the possibility to import/export coordinates of atoms in text format can be easily used. Chemcraft itself does not perform calculations, but can significantly facilitate the use of widespread quantum chemistry packages. Chemcraft runs under Windows and Linux (but the Linux version has some disadvantages).
ChemDraw	https://perkinelmerinformatics.com/products/research/chemdraw/	ChemOffice includes ChemDraw. ChemDraw® solutions have provided capabilities and integrations to help to turn ideas and drawings into publications.
Chemical shift index	https://link.springer.com/referenceworkentry/10.1007%2F978-3-642-16712-6_317	The chemical shift index or CSI is a simple graphical method that can be used to display and identify the type, location, and extent of secondary structures (helices, beta strands, and random coil regions) in polypeptides using only backbone chemical shift information.
Chemical WorkBench	http://www.kintechlab.com/products/chemical-workbench/	Chemical WorkBench is a software for kinetic mechanism analysis and device/reactor scale kinetic modeling of processes in gases, plasma and at the gas-solid interfaces. The primary users are researchers and engineers, involved into kinetic models development as well as thermodynamic and kinetic modeling for chemical engineering, combustion, catalysis, metallurgy and microelectronics areas.
Chemicalize	https://chemicalize.com/welcome	Chemicalize calculates properties instantly via structure-based predictions, allows structure-based search through patents and journals and draw publication-ready structures.
Chemis3D	http://en.bio-soft.net/3d/chemis3d.html	It is not working. Chemis3D is a Java Applet which renders virtual 3D molecular models within a Web document
Chemistry Development Kit	https://cdk.github.io/	The Chemistry Development Kit (CDK) consists on a collection of modular Java libraries for processing chemical data. It includes molecules and reaction valence bond representation, efficient molecule processing algorithms, coordinate generation and rendering…

ChemOffice	http://www.cambridgesoft.com/Ensemble_for_Chemistry/details/Default.aspx?fid=16	ChemOffice is a scientifically intelligent, integrated suite of personal productivity tools that helps scientists to efficiently keep track of their work, gain a deeper understanding of their data and produce scientific reports professionally and efficiently. This software includes ChemDraw, Chem3D and ChemFinder.
Chemsketch	https://www.acdlabs.com/products/draw_nom/draw/chemsketch/	ACD/ChemSketch is an easy-to-use, chemically intelligent molecular structure drawing application, with more than 2 million users worldwide. Draw chemical structures, reactions, and schema, and access a graphical tools and templates. Generate names from molecular structure, calculate molecular properties from chemical structure, create reports, presentations, and publication-ready figures.
ChemSpider (JS-mol)-Web	http://www.chemspider.com/	ChemSpider is a free chemical structure database providing fast text and structure search access to over 100 million structures from hundreds of data sources.
ChemVLab+ WEB	https://www.modelscience.com/products.html	Model ChemLab is a unique product incorporating both an interactive simulation and a lab notebook workspace with separate areas for theory, procedures and student observations. Commonly used lab equipment and procedures are used to simulate the steps involved in performing an experiment. Users step-through the actual lab procedure while interacting with animated equipment in a way that is similar to the real lab experience.
ChemWindow	https://sciencesolutions.wiley.com/chemwindow-chemical-structure-drawing-software/	ChemWindow Edition is the software chemists choose for chemical structure drawing and publishing worldwide, with integrated solutions to modify, store, search, and retrieve chemical structures and properties.
Chem-X	https://pubmed.ncbi.nlm.nih.gov/8366147/	3D structure generation program.
CHIME	http://www.chemistry.wustl.edu/~edudev/chime.html	The Chime plug-in was developed by MDL Information Systems, Inc. It displays 2D and 3D molecules directly within a web page and works with both Netscape and Microsoft browsers. The molecules in the web page are "live," meaning they are not just pictures, but chemical structures that can be rotated, reformatted, and saved in various file formats for use in modeling or database software.
Chimera	https://www.cgl.ucsf.edu/chimera/	UCSF Chimera is a program for the interactive visualization and analysis of molecular structures and related data, including density maps, trajectories, and sequence alignments. It is available free of charge for noncommercial use.
Cn3D	https://www.ncbi.nlm.nih.gov/Structure/CN3D/cn3d.shtml	Cn3D (3D) is a helper application for your web browser that allows you to view 3D structures from NCBI's Entrez Structure database. Cn3D is provided for Windows and Macintosh, and can be compiled on Unix. Cn3D simultaneously displays structure, sequence, and alignment, and now has powerful annotation and alignment editing features.
COLUMBUS	https://www.univie.ac.at/columbus/	COLUMBUS is a collection of programs for high-level ab initio molecular electronic structure calculations. The programs are designed primarily for extended multi-reference (MR) calculations on electronic ground and excited states of atoms and molecules
Composer	https://pubmed.ncbi.nlm.nih.gov/2099735/	It is not working. A computer program for modelling proteins which uses 3D structures defined by X-ray analysis together with rules defined by their analysis and comparison.
Computational Center Macromolecular S.	https://www.sdsc.edu/CCMS/	The Computational Center for Macromolecular Structure (CCMS) is distributing and supporting software for the determination and analysis of the structures of biological macromolecules.
CONCORD	http://mw.concord.org/nextgen/	It is into Molecular Workbench (MW), this is a software that provides visual, interactive computational experiments for teaching and learning science.
CONQUEST	https://www.ccdc.cam.ac.uk/solutions/csd-core/components/conquest/	ConQuest provides advanced 3D searching of structures in the Cambridge Structural Database. ConQuest is the primary program for searching and retrieving information from the Cambridge Structural Database (CSD). Local in-house databases generated using PreQuest can also be searched alongside the CSD using ConQuest.
Coot Crystallographic Object-Oriented Toolkit	https://www2.mrc-lmb.cam.ac.uk/personal/pemsley/coot/ http://bernhardcl.github.io/coot/ (wincoot)	A graphics program for building, refining and analyzing macromolecular models obtained with crystallographic procedures. Coot is for macromolecular model building, model completion and validation, particularly suitable for protein modelling using X-ray data. Coot displays maps and models and allows model manipulations such as idealization, real space refinement, manual rotation/translation, rigid-body fitting, ligand search, solvation, mutations, rotamers, Ramachandran plots, skeletonization, and non-crystallographic symmetry.
COSILAB	https://www.rotexo.com/index.php/en/	Combustion Simulation Laboratory for the numerical simulation of tasks in the areas of combustion and reactive flows.
CP2K	http://ccs-psi.org/node/2	CP2K is a free, open-source quantum chemistry software package designed to perform molecular dynamics and Monte Carlo simulations of clusters and periodic systems. CP2K can be run in both MPI and OpenMP modes, and built-in farming procedures allow for capacity jobs at DOE Leadership Computing Facilities.
Crystal	https://www.crystal.unito.it/features.php	A computational tool for solid state chemistry and physics.
CrystalMaker	http://crystalmaker.com/	Build, display and manipulate crystal and molecular structures. To design new materials and relax their structures; animate structural behaviour; generate video for teaching or presentations; simulate diffraction properties for powders and single crystals.
CS23D	http://www.cs23d.ca/	A web server for generating accurate 3D protein structures using only assigned NMR chemical shifts as input.
CS ChemOffice Pro	https://onlinelibrary.wiley.com/doi/pdf/10.1002/ejtc.20	Chem3D is part of the CS ChemOffice package. It comprises three parts: ChemDrawPro(a 2D drawing program); Chem3DPro (which converts 2D structures into 3D models) and ChemFinder Pro (which the vendors describe as ‘organizational software’).

Cuemol	http://www.cuemol.org/	CueMol's aims are to visualize and create the publication-quality images of the macromolecular structures with user-friendly interfaces. Currently supported files are molecular coordinates (PDB format), electron density (CCP4, CNS , and MTZ formats), MSMS surface data, and APBS electrostatic potential map (OpenDX format).
Chemitorium	https://sourceforge.net/projects/chemitorium/	Analysis and Visualisation Tool; Chemical formula editor, calculation of threedimensional molecular structures, high-quality realtime rendering.
Dalton	https://daltonprogram.org/	Calculations of molecular properties at the HF, DFT, MCSCF, MC-srDFT, and CC levels of theory.
DelPhi	http://compbio.clemson.edu/delphi	Calculates electrostatic potentials in and around macromolecules and the corresponding electrostatic energies. It incorporates the effects of ionic strength mediated screening by evaluating the Poisson-Boltzmann equation at a finite number of points within a 3D grid box. DelPhi is commonly used in protein science to visualize variations in electrostatics along a protein or other macromolecular surface and to calculate the electrostatic components of various energies. Modeling the electrostatic potential of biological macromolecules is that they exist in water at a given ionic strength and that they have an irregular shape.
DGEOM 95	…	It is not working.
DGII	…	It is not working.
Dirac	http://www.diracprogram.org/doku.php	It computes molecular properties using relativistic quantum chemical methods.
DISCO	…	It is not working.
Discover	…	It is not working.
Discovery Studio	https://www.discngine.com/discovery-studio	Comprehensive, collaborative modeling and simulation application for Life Sciences discovery research. Simulating small molecule and macromolecule systems. It makes use of a number of software algorithms developed originally in the scientific community, including CHARMM, MODELLER, DELPHI, ZDOCK, DMol3 and more.
DMol3	https://www.swmath.org/software/6115	DMol3 is a unique, accurate, and reliable density functional theory (DFT) quantum mechanical code for research in the chemicals and pharmaceutical industries. It uses density functional theory with a numerical radial function basis set to calculate the electronic properties of molecules, clusters, surfaces and crystalline solid materials from first principles.
DockingServer WEB	https://www.dockingserver.com/web	Web-based interface that handles all aspects of molecular docking from ligand and protein set-up.
DP code	http://www.dp-code.org/	Linear Response Time-Dependent Density-Functional Theory (LR-TDDFT) code in Frequency Reciprocal (k-ω) space on a Plane Waves (PW) basis set. Some quantities (the Kohn-Sham polarizability and the exchange correlation kernel) are calculated in Real(r) space. The purpose: Dielectric and Optical Spectroscopy (Optical Absorption, Reflectivity, Refraction Indices, EELS, IXSS, CIXS...).
Drug Bank WEB	https://drugbank.com/	Online database containing information on drugs and drug. DrugBank combines detailed drug (i.e. chemical, pharmacological and pharmaceutical) data with comprehensive drug target (i.e. sequence, structure, and pathway) information.
DSSP	https://swift.cmbi.umcn.nl/gv/dssp/	A database of secondary structure assignments for all protein entries in the Protein Data Bank (PDB). DSSP is also the program that calculates DSSP entries from PDB entries.
EGO	http://www-s.ks.uiuc.edu/Development/biosoftdb/biosoft.cgi?application=238&category=2	EGO is a program to perform molecular dynamics simulations on parallel as well as on sequential computers.
enCIFer	https://www.ccdc.cam.ac.uk/Community/csd-community/encifer/	Visualise structure(s) in a CIF, including displacement ellipsoids. The Crystallographic Information File (CIF) is the internationally agreed standard file format for information exchange in crystallography.
ePMV	http://epmv.scripps.edu/	uPy plugin, embedded Python Molecular Viewer (ePMV) runs molecular-modeling software directly in 3D animation applications (hosts).
Eulim	https://github.com/ilm-labs/ilm	Chemistry library written in Ruby under the MIT license. Eulim is a Ruby gem for Chemistry, which supports the calculation of molecular mass of compound, balancing chemical equations, efficient handling of states of chemical species and many more things.
EXC code	…	It is not working. EXC is a condensed matter physics many-body theory software package implementing the Bethe–Salpeter equation in frequency-reciprocal space and on a plane wave basis set. Its purpose is to calculate, ab initio, dielectric and optical properties, like absorption, reflectivity, refraction index, electron and X-ray energy loss, for a large variety of systems, ranging from bulk systems, surfaces, to clusters or atoms. It is distributed under the GNU/GPL license.
Extensible Computational Chem.	…	It is not working. A graphical user interface, scientific visualization tools, and the underlying data management framework enabling scientists to efficiently set up calculations and store, retrieve, and analyze the rapidly growing volumes of data produced by computational chemistry studies.
EzMol Web	http://www.sbg.bio.ic.ac.uk/ezmol/	Molecular modeling web server for the visualization of protein molecules.

FDMNES	http://fdmnes.neel.cnrs.fr/	It calculates the spectra of different spectroscopies related to the real or virtual absorption of x-ray in material. It gives the absorption cross sections of photons around the ionization edge, that is in the energy range of XANES. The calculation is performed with all conditions of rectilinear or circular polarization.
FHI-aims	https://aimsclub.fhi-berlin.mpg.de/	Initio molecular simulations is a shared-source software package for computational molecular and materials science written in Fortran. It uses density functional theory and many-body perturbation theory to simulate chemical and physical properties of atoms, molecules, nanostructures, solids, and surfaces.
Firefly	http://classic.chem.msu.su/gran/gamess/index.html	Ab initio computational chemistry program for Intel-compatible x86, x86-64 processors based on GAMESS (US) sources. However, it has been mostly rewritten (60-70% of the code), especially in platform-specific parts (memory allocation, disk input/output, network), mathematic functions (e.g., matrix operations), and quantum chemistry methods (such as Hartree–Fock method, Møller–Plesset perturbation theory, and density functional theory).
Foldit	https://fold.it/	Not interesting here.
FreeON		An experimental, open source (GPL) suite of programs for linear scaling quantum chemistry, formerly known as MondoSCF.
Gabedit	http://gabedit.sourceforge.net/	Gabedit is a graphical user interface to computational chemistry packages like Gamess-US, Gaussian, Molcas, Molpro, MPQC, OpenMopac, Orca, PCGamess and Q-Chem. It can display a variety of calculation results including support for most major molecular file formats. The advanced "Molecule Builder" allows to sketch in molecules and examine them in 3D. Graphics can be exported to various formats, including animations.
GALAXY	http://galaxy.seoklab.org/	A scientific workflow, data integration, and data and analysis persistence and publishing platform that aims to make computational biology accessible to research scientists that do not have computer programming or systems administration experience.
GAMESS	https://www.msg.chem.iastate.edu/gamess/	A general ab initio quantum chemistry package.
GASP	https://www.aerosoftinc.com/gasp/features_main.php	Software for geometric simulations of flexibility in polyhedral and molecular framework structures
Gaussian- GausView	https://gaussian.com/	Purpose computational chemistry software package. The name originates from Pople's use of Gaussian orbitals to speed up molecular electronic structure calculations as opposed to using Slater-type orbitals, a choice made to improve performance on the limited computing capacities of then-current computer hardware for Hartree–Fock calculations.
GEM	https://gems.web.psi.ch/	Geochemical Modeling, code for solving for geochemical equilibria (with TSolMod library), generic parameter-fitting code coupled with the GEMS3K code, and default chemical thermodynamic database of GEM-Selektor package
GeNMR	http://www.genmr.ca/index.php	Web server for generating 3D protein structures using NOE-derived distance restraints and NMR chemical shifts. The web server produces an ensemble of PDB coordinates within a period ranging from 20 minutes to 4 hours. Template-based method of protein structure determination that utilizes both NMR chemical shifts and NOE-based distance restraints.
GERM	https://earthref.org/GERM	Geochemical Earth Reference Model. Chemical characterization of the Earth, its major reservoirs and the fluxes between them.
Ghemical-GMS	https://www.bioinformatics.org/ghemical/ghemical/index.html	Ghemical is computational chemistry package, which is licensed under GNU GPL. Ghemical is implemented using the C++ programming language, and it has a graphical user interface which utilizes the OpenGL graphics interface and the GTK+ multiplatform widget library.
GLARE	http://glare.sourceforge.net/	It facilitates and improves the design of chemical combinatorial libraries. This program reduces or eliminates the time a combinatorial chemist spends examining reagents which a priori cannot be part of a 'good library'. The principal objective of the algorithm behind GLARE is to provide a combinatorial set of virtual products that satisfies user defined filtering rules.
Glauber	https://en.wikipedia.org/wiki/Glauber	Not interesting here.
GNU Octave	https://www.gnu.org/software/octave/index	High-level programming language, primarily intended for numerical computations. Mathematics-oriented syntax with built-in 2D/3D plotting and visualization tools. Octave helps in solving linear and nonlinear problems numerically, and for performing other numerical experiments using a language that is mostly compatible with MATLAB.
gOpenMol	https://www.ch.cam.ac.uk/computing/software/gopenmol	A molecular visualization program, visualizing molecular orbitals, electron densities, spin densities, etc.
GPCR-ModSim	http://gpcr-modsim.org/	A pipeline for computational modeling and simulation of G-Protein Coupled Receptors.
Graffiti		It is not working.
GRAMM	https://vakserlab.ku.edu/resources_gramm1.03.php	A program for protein docking. To predict the structure of a complex, it requires only the atomic coordinates of the two molecules (no information about the binding sites is needed). The program performs an exhaustive 6-dimensional search through the relative translations and rotations of the molecules.
GRAMM-X	http://vakser.compbio.ku.edu/resources/gramm/grammx	Protein-Protein Docking Web Server. You can submit input files and parameters to this web server and the docking simulation will be run on our computer cluster.

GRASP	http://honig.c2b2.columbia.edu/grasp	(Graphical Representation and Analysis of Structural Properties) Gromacsused by the structural biology community to visualize macromolecules. Its particular strengths compared to other such programs is its facility with surfaces and with electrostatics.
GROMACS	https://www.gromacs.org/	A molecular dynamics package mainly designed for simulations of proteins, lipids, and nucleic acids.
GROMOS	http://www.gromos.net/	GROningen MOlecular Simulation computer program package, a force field for molecular dynamics simulation, and a related computer software package.
HASL	…	It is not working.
hBar Lab	…	It is not working.
HBPLUS	https://www.ebi.ac.uk/thornton-srv/software/HBPLUS/	To compute hydrogen positions, hydrogen bonds, and neighboring interactions.
Hingefind Algorithm	https://biomachina.org/disseminate/hingefind/hingefind.html	Algorithm to investigate domain motions in proteins http://www.ccl.net/cca/software/SOURCES/FORTRAN/hingefind/hingefind.html
HINT!	http://www.edusoft-lc.com/hint/	(Hydropathic INTeractions) Empirical molecular modeling system with new methods for de novo drug design and protein or nucleic acid structural analysis. hint! calculates 3D hydropathic interaction maps that are uniquely instructive for understanding biomacromolecular structure: substrate/inhibitor/drug binding to proteins and nucleotides, protein subunit interactions, and protein folding.
HOLLOW	…	It is not working.
HONDO	https://comp.chem.umn.edu/hondoplus/	It is not working.
HORTON	https://theochem.github.io/horton/2.0.1/index.html	Helpful Open-source Research TOol for N-fermion systems, is an open-source modular quantum chemistry program written primarily in Python. It is composed of several quantum mechanical methods for electronic structure calculations and tools for post-processing wave functions and densities.
HyperChem Hypercube	https://www.chemits.com/en/software/molecular-modeling/hyperchem/	Computational methods include molecular mechanics, molecular dynamics, and semi-empirical and ab-initio molecular orbital methods, as well as density functional theory. HyperChem Data and Hyper NMR are included as part of HyperChem. This software is applicable to macromolecules as well as small molecules and is scriptable. www.hipercubeusa.com
ICM Chemist (Molsoft)	http://www.molsoft.com/icm-chemist.html	A standalone suite of programs for chemical drawing and editing, chemical database generation, chemical searching, clustering, and enumeration.
ICM-Browser (Molsoft)	http://www.molsoft.com/icm_browser.html	Molecule visualization, fully interactive 3D slides in powerpoint and web, publication quality images, display ligand binding pocket surfaces, hydrogen bond display, measure distances and angles.
IcmJS (Molsoft)	https://www.molsoft.com/activeicmjs.html	A JavaScript/HTML5 3D molecular viewer which does not require any plug-in or browser extension and runs inside any modern browser. IcmJS brings desktop quality graphics to the web applications.
Iditis	…	It is not working
IMol	https://www.pirx.com/iMol/index.shtml	A molecular visualization application for Mac OS X operating system, for chemists and molecular biologists.
Insight II	http://www.serc.iisc.ac.in/software/insight-ii/	Molecular modelling codes specifically designed for biological systems, to create, display, and rotate 3D chemical structures with built-in support for proteins and nucleic acids. The structures can be selectively colored and rendered for aiding analysis.
Internal Coordinate Mechanics	…	Not interesting here.
ISIS Draw	https://isis-draw-for-windows.software.informer.com/2.5/	A chemical structure drawing program developed by MDL Information Systems. It introduced a number of file formats for the storage of chemical information that have become industry standards.
Iview WEBGL	…	A visualizer for protein-ligand complex.
Jaguar	https://www.schrodinger.com/products/jaguar	A computer software package used for ab initio quantum chemistry calculations for both gas and solution phases.
JChemPaint	https://jchempaint.github.io/	the editor and viewer for 2D chemical structures developed using the Chemistry Development Kit (CDK).
Jmol JavaScript-Based Molecular	https://launchpad.net/jmol	Computer software for molecular modelling chemical structures in 3D.
JOELib	ra.cs.uni-tuebingen.de/software/joelib/introduction.html	Platform independent open source computational chemistry package written in Java. Computer software, a chemical expert system used mainly to interconvert chemical file formats. Because of its strong relationship to informatics, this program belongs more to the category cheminformatics than to molecular modelling.
Joint Expert Speciation System JESS	http://jess.murdoch.edu.au/jess_home.htm	Package of computer software and data developed collaboratively at Murdoch University and elsewhere by researchers interested in the chemical thermodynamics of water solutions with important applications in industry, biochemistry, medicine and the environment.
Jolecule Protein viewer	https://jolecule.appspot.com/	Viewer for proteins and DNA designed for making animated slideshows. It runs on web-browsers using WebGL via the three.js library.

JSME/JME Molecule Editor	https://jsme-editor.github.io/	A molecule editor Java applet with which users make and edit drawings of molecules and reactions (including generating substructure queries), and can display molecules within an HTML page. The editor can generate Daylight simplified molecular-input line-entry system (SMILES) or MDL Molfiles of the created structures.
Kekulé Program	http://partridgejiang.github.io/Kekule.js/	Open source JavaScript library for chemoinformatics released under MIT license.
Khimera	http://www.kintechlab.com/products/khimera/	Khimera is used to calculate the kinetic parameters of microscopic processes, thermodynamic and transport properties of substances and their mixtures in gases, plasmas and gas-solid phases boundary. It is a software for calculation of the kinetic parameters of microscopic processes, thermodynamic and transport properties of substances and their mixtures in gases, plasmas and also of heterogeneous processes.
Kinemage, MAGE & King	http://kinemage.biochem.duke.edu/	An interactive graphic scientific illustration. It often is used to visualize molecules, especially proteins although it can also represent other types of 3-dimensional data (such as geometric figures, social networks, or tetrahedra of RNA base composition). Mage displays the 3D relationships between data in an interactive environment which facilitates both open-ended exploration and structured presentation. It includes many tools for on-screen measurement, construction, and editing of the display objects; it can write either Kinemage output or various types of 2D image output.
Kinetic PreProcessor KPP	http://people.cs.vt.edu/~asandu/Software/Kpp/	An open-source software tool used in atmospheric chemistry; taking a set of chemical reactions and their rate coefficients as input, KPP generates Fortran 90, FORTRAN 77, C, or Matlab code of the resulting ordinary differential equations (ODEs); solving the ODEs allows the temporal integration of the kinetic system.
Leapfrog	…	LeapFrog generates new compounds by repeatedly making small structural changes, rapidly evaluating the binding energy of the new compound, and keeping or discarding the changes based on the results.
LigPlot	https://www.ebi.ac.uk/thornton-srv/software/LigPlus	Biogchemistry. Automatically generates schematic diagrams of protein-ligand interactions for a given PDB file.
LiSiCA	http://insilab.org/lisica/	LiSiCA (Ligand Similarity using Clique Algorithm) is a ligand-based virtual screening software that searches for 2D and 3D similarities between a reference compound and a database of target compounds which should be represented in a Mol2 format.
List of protein-ligand dockings	…	AutoDock and AutoDock Vina, rDock, FlexAID, Molecular Operating Environment, and Glide. Biochemistry.
Look/GeneMine	www.bioinformatics.ucla.edu/genemine	It is not working.
Loopy	http://honig.c2b2.columbia.edu/loopy	Biochemistry – 3D Structural prediction. Loopy is a program for predicting protein loop conformations or segment mutation.
Ludi		It is not working. Biochemistry – Protein-small molecule interaction. Drug design
Luscus	https://sourceforge.net/projects/luscus/	Luscus can visualize dipole moments, normal modes, molecular orbitals, electron densities and electrostatic potentials.
MacroModel	https://www.schrodinger.com/products/macromodel	Molecular dynamics simulations. Force field-based molecular modeling is routinely applied to examine molecular conformations, molecular motion, and intermolecular interactions for a wide range of different materials including organic and inorganic molecules and oligomers, organometallic complexes as well as complex biological systems.
MADNESS	https://github.com/m-a-d-n-e-s-s/madness	Quantum mechanics. MADNESS (Multiresolution Adaptive Numerical Environment for Scientific Simulation) is a high-level software environment for the solution of integral and differential equations in many dimensions using adaptive and fast harmonic analysis methods with guaranteed precision based on multiresolution analysis and separated representations .
MAGE	http://kinemage.biochem.duke.edu/software/mage.php	It is included into Kinemage, MAGE & King. This is a model building, used in both teaching and research, in applications ranging from estuary ecology to X-ray crystallography model quality assessments, Mage displays the 3D relationships between data in an interactive environment which facilitates both open-ended exploration and structured presentation. It includes many tools for on-screen measurement, construction, and editing of the display objects; it can write either Kinemage output or various types of 2D image output
Marvin	https://chemaxon.com/products/marvin	Marvin suite is a chemically intelligent desktop toolkit built to help you draw, edit, publish, render, import and export your chemical structures and as well as allowing you to convert between various chemical and graphical file formats. It is free for individual, academic and non-commercial use.
Match	https://www.crystalimpact.com/match/	Crystallography. Match! is an easy-to-use software for phase analysis using powder diffraction data. It compares the diffraction pattern of your sample to a database containing reference patterns in order to identify the phases which are present. Additional knowledge about the sample like known phases, elements or density can be applied easily.
MC-SYM	https://www.major.iric.ca/MC-Sym/	Model building, Biochemistry. The MC-Fold/MC-Sym pipeline is a web-hosted service for RNA secondary and tertiary structure prediction. The pipeline means that the input sequence to MC-Fold outputs secondary structures that are direct input to MC-Sym, which outputs tertiary structures.
MDL Chime	https://www.umass.edu/microbio/chime/getchime.htm	Molecular Visualization Resources.

MembraneEditor CELLmicrocosmos	https://www.cellmicrocosmos.org/index.php/cm2-project	A modular interactive shape-based software approach to solve heterogeneous membrane packing problems
Mercury Crystal Structure Visual	https://www.ccdc.cam.ac.uk/solutions/csd-core/components/mercury/	Crystallography. Mercury offers a comprehensive range of tools for 3D structure visualization, the exploration of crystal packing and the statistical analysis of CSD search data.
MGLTools (Python & AutoDock)	http://mgltools.scripps.edu/	MGLTools is a software developed at the Molecular Graphics Laboratory (MGL) of The Scripps Research Institute for visualization and analysis of molecular structures.
Midas		Not interesting here.
MM3	http://www.colby.edu/chemistry/PChem/scripts/wMM3.html	Molecular mechanics MM. Create an input file for MM3 Molecular Mechanics Calculations.
MODELLER	https://salilab.org/modeller/	Biochemistry, model building. MODELLER is used for homology or comparative modeling of protein three-dimensional structures
Moil	https://biohpc.cornell.edu/software/moil/moil.html	Molecular dynamics, modeling. Free software for molecular dynamics and modeling.
Mol2Mol	https://www.gunda.hu/mol2mol/index.html	Model building, molecular representations.
MOLCAS	https://www.molcas.org/	Quantum chemistry. Molcas is an ab initio quantum chemistry software package. The basic philosophy is to be able to treat general electronic structures for molecules consisting of atoms from most of the periodic table. As such, the primary focus of the package is on multi-configurational methods with applications typically connected to the treatment of highly degenerate states.
Molconn-z	http://www.edusoft-lc.com/molconn/	Model building, biochemistry. Molconn-Z is the standard program for generation of Molecular Connectivity, Shape, and Information Indices for Quantitative Structure Activity Relationship (QSAR) Analyses. New parameters and concepts of QSAR, including the E-State, have been introduced first in Molconn-Z.
Molden UNIX	https://www3.cmbi.umcn.nl/molden/howtoget.html	Quantum chemistry for molecular visualization.
Moldraw	https://iris.unito.it/handle/2318/42993	Molecular modelling; molecular visualizer; crystal structure viewer.
Molecular operating environment	https://www.chemcomp.com/Products.htm	Integrated Computer-Aided Molecular Design Platform Small Molecules - Peptides – Biologics.
Molecular Rift	…	Tool for Virtual Reality.
Molecular Workbench-Concord	http://mw.concord.org/modeler/	Molecular visualization, only for teaching.
Molegro Virtual Docker	http://molexus.io/molegro-virtual-docker/	Biochemistry, docking. Molegro Virtual Docker is an integrated platform for predicting protein – ligand interactions.
MOLEKEL	https://ugovaretto.github.io/molekel/	Molekel is an open-source multi-platform molecular visualization program.
MOLGEN	https://www.molgen.de/online.html	Molecular visualization.
MolPOV2	https://richardson.chem.ufl.edu/software/molpov2-download/	Molecular representation, Biochemistry, protein data-base. MolPOV is a graphics file converter that reads Protein Data Bank format files and produces a ready-to-render input file for the Persistence of Vision ray tracer (POV-Ray)
MolPro	https://www.molpro.net/	Quantum chemistry. Molpro is a comprehensive system of ab initio programs for advanced molecular electronic structure calculations
MolScript	https://kraulis.se/MolScript/	Molecular representation. MolScript is a program for creating schematic or detailed molecular graphics images from molecular 3D coordinates, usually, but not exclusively, protein structures.
Molsketch	https://sourceforge.net/projects/molsketch/	Molsketch is a 2D molecular editing tool.
MoluCAD	https://www.kinematics.com/products/molucad.php	Molecular mechanics, molecular representation, special for education. MoluCAD is a full-featured molecular modeling and visualization tool designed for Windows.
MolView	https://molview.org/	Molecular representation, special for education. MolView is an Open-Source web-application to make science and education. MolView is mainly intended as web-based data visualization platform.
MOPAC	http://openmopac.net/	Quantum chemistry, molecular representation. MOPAC (Molecular Orbital PACkage) is a semiempirical quantum chemistry program based on Dewar and Thiel's NDDO approximation.
MOPLOT	http://moplot.sourceforge.net/	Quantum mechanics, molecular orbitals visualization. MOPLOT fills this gap by producing 2-D projected images of geometries, MO's and normal vibrations produced immediately from the output of various standard quantum chemical programs.
MovieMol	…	It is not working.
MPQC	https://mpqc.org/	Quantum chemistry. Massively Parallel Quantum Chemistry (MPQC) platform is a research package for ab initio simulation of the electronic structure of molecules and periodic solids, with primary focus on many-body electronic structure methods, such as coupled-cluster.

NAMD Molecular visualizations	http://www.ks.uiuc.edu/Research/namd/	Model building, visualization, biochemistry. NAMD is a parallel molecular dynamics code designed for high-performance simulation of large biomolecular systems.
NAMOT	http://namot.sourceforge.net/	Molecular visualization of nucleic acids. Nucleic Acid Modeling Tool (NAMOT) is a program to allow users to manipulate molecular models of nucleic acids.
Nanome PC	https://nanome.ai/	Global real-time collaboration using Virtual Reality from general chemistry to pharmaceutical drug discovery. Visualize docked results; analyze the 3D arrangement of molecules while inside a protein binding pocket.
NAOMI/Unicon	https://www.zbh.uni-hamburg.de/en/forschung/amd/software/naomi.html	Model building, biochemistry, drug design. NAOMI is a command-line tool for the consistent conversion of common chemical file formats (SDF, SMILES, MOL2). It is based on a chemical model which is designed to describe organic molecules relevant in the context of drug discovery.
Newton-X	https://newtonx.org/	Quantum chemistry, semi-empirical methods. NX is a general-purpose program package for simulating the dynamics of electronically excited molecules and molecular assemblies. It is a platform for performing all steps of the simulation, from the generation of the initial conditions to the statistical analysis of the results.
NGL viewer molecules	https://nglviewer.org/	Molecular representation. NGL Viewer is a collection of tools for web-based molecular graphics. WebGL is employed to display molecules like proteins and DNA/RNA with a variety of representations.
NWChem	https://www.nwchem-sw.org/	From quantum to classical, and all combinations. NWChem aims to provide its users with computational chemistry tools that are scalable both in their ability to treat scientific computational chemistry problems, and in their use of available parallel computing resources from high-performance parallel supercomputers to conventional workstation clusters.
OctaDist	https://octadist.github.io/	3D modelling of complex. OctaDist (Octahedral Distortion calculator) is an inorganic chemistry and crystallography program for computing the distortion parameters, such as distance and angle distortions, in coordination complexes.
Octopus	https://octopus-code.org	Molecular representation, ab initio, quantum chemistry. Octopus is a scientific program aimed at the ab initio virtual experimentation on a hopefully ever-increasing range of system types. Electrons are described quantum-mechanically within density-functional theory (DFT), in its time-dependent form (TDDFT) when doing simulations in time. Nuclei are described classically as point particles.
OELib	it is not working	Obsolete.
ONETEP	https://www.onetep.org/	Molecular representation, Quantum mechanics. ONETEP (Order-N Electronic Total Energy Package) is a linear-scaling code for quantum-mechanical calculations based on density-functional theory.
Open Babel	http://openbabel.org/wiki/Main_Page	Molecular mechanics, visualization. Open Babel is a chemical toolbox designed to speak the many languages of chemical data. It's an open, collaborative project allowing anyone to search, convert, analyze, or store data from molecular modeling, chemistry, solid-state materials, biochemistry, or related areas.
OpenAtom	http://charm.cs.illinois.edu/OpenAtom/	Ab-initio molecular dynamics. OpenAtom is a software for studying atomic, molecular, and condensed phase materials systems based on quantum chemical principles.
ORCA Quantum Chemistry Prog.	https://www.faccts.de/orca/	Quantum chemistry. ORCA has more than 15.000 registered users in academia worldwide, ORCA is a quantum-chemical software package to date. It provides cutting-edge methods in the fields of Density Functional Theory as well as correlated wave-function based methods.
OSRA	https://cactus.nci.nih.gov/osra/	OSRA is a utility designed to convert graphical representations of chemical structures, as they appear in journal articles, patent documents, textbooks, trade magazines etc., into SMILES (Simplified Molecular Input Line Entry Specification) or SD files.
PARSEC	https://parsec.oden.utexas.edu/	PARSEC is a computer code that solves the Kohn-Sham equations by expressing electron wave-functions directly in real space, without the use of explicit basis sets. It uses norm-conserving pseudopotentials (Troullier-Martins and other varieties). It is designed for ab initio quantum-mechanical calculations of the electronic structure of matter, within density-functional theory.
PCModels	https://www.daylight.com/products/pcmodels.html	PCModels is a Daylight software module providing access to two chemical models: CLOGP (hydrophobicity partition coefficient) and CMR (molar refractivity).
PDB Protein Data Bank	https://www.rcsb.org/	DB, biochemistry, molecular representation. Protein Data Bank archive-information about the 3D shapes of proteins, nucleic acids, and complex assemblies that helps students and researchers understand all aspects of biomedicine and agriculture, from protein synthesis to health and disease.
Perse Visualizer	https://apps.apple.com/es/app/perse-visualizer/id1476999836	Mobile protein visualizer. Search for any protein from the RCSB protein data bank by name or ID.
PLATO	https://www.imperial.ac.uk/people/a.horsfield/research.html	Crystallography (also for molecules). Quantum chemistry, solids. PLATO (Package for Linear-combination of ATomic Orbitals) is a suite of programs for electronic structure calculations. It receives its name from the choice of basis set (numeric atomic orbitals) used to expand the electronic wave functions.

PocketAnalyzerPCA	https://sourceforge.net/projects/papca/	PocketAnalyzerPCA combines a geometric algorithm for detecting pockets in proteins with Principal Component Analysis and clustering. This enables visualization and analysis of pocket conformational distributions of large sets of protein structures.
POLYVIEW-3D	http://polyview.cchmc.org/polyview3d.html	POLYVIEW-3D is a web-based tool for macromolecular structure visualization and analysis. In particular, it provides a wide array of options for automated structural and functional analysis of proteins and their complexes.
PovChem	https://www.chemicalgraphics.com/PovChem/	Representation of molecules. It's a chemical visualization and illustration program with a new graphic interface. It takes molecules in the PDB format, lets you to set up a picture with fine control over details of the illustration-colors, atom and bond radii, view orientation, etc.
PQS 3D stereo systems	http://www.pqs-chem.com/index.php	Molecular representation. 3D stereo enabled line of workstations. The stereo effect is achieved through the use of active shutter glasses operating at 120Hz refresh rate. The glasses are synchronized with the display, either a projector/monitor, producing two distinct images, one for each eye. The effect is far superior to the earlier technologies of passive polarized and anaglyph (red/blue) glasses.
ProBiS333	…	It is not interesting here.
Profix	…	It is not interesting here.
PrologD	…	It is not interesting here.
PrologP	…	It is not interesting here.
PROSA II	https://prosa.services.came.sbg.ac.at/prosa.php	Protein structure analysis.
Prosat+	https://prosat.h-its.org/	ProSAT+ is a tool to explore the relation between sequence and structural properties. The structure is displayed using JSmol, a JavaScript implementation of Jmol (Java plugins). ProSAT+ allows the visualization of a 3D protein structure with highlighted functional regions together with annotations of the functional effects of point mutations.
Protein Explorer	http://www.biorom.uma.es/contenido/pe/protexpl/frntdoor.htm	Exploration DNA-protein.
PSI	https://psicode.org/	Open-Source Quantum Chemistry.
Psi88	…	Obsolete.
PubChem 3D	https://pubchemdocs.ncbi.nlm.nih.gov/pubchem3d	Molecular representation. The PubChem3D project computes a 3-D description of PubChem Compound records.
PV-JavaScript Protein Viewer	https://biasmv.github.io/pv/	Molecular representations. PV is a JavaScript viewer to visualize protein structures directly in the browsers.
PyMol	https://pymol.org/2/	Molecular representations. PyMOL is a user-sponsored molecular visualization system on an open-source foundation, maintained and distributed by Schrödinger.
PyQuante	http://pyquante.sourceforge.net/	Quantum chemistry. PyQuante (Sourceforge Project Page) is an open-source suite of programs for developing quantum chemistry methods.
PySCF	https://pyscf.org/	Quantum chemistry calculations. The Python-based Simulations of Chemistry Framework (PySCF) is an open-source collection of electronic structure modules powered by Python.
Python Molecular Viewer PMV	http://mgltools.scripps.edu/documentation/tutorial/python-molecular-viewer	Molecular representations.
Qbox	http://qboxcode.org/	Molecular dynamics. Qbox is a C++/MPI scalable parallel implementation of first-principles molecular dynamics (FPMD) based on the plane-wave, pseudopotential formalism.
Q-Chem	https://www.q-chem.com/	Quantum chemistry, molecular representations. Q-Chem is a comprehensive ab initio quantum chemistry software for accurate predictions of molecular structures, reactivity, and vibrational, electronic and NMR spectra.
QUANTA	…	It is not interesting here.
Quantemol-DB	…	It is not interesting here.
Quantum chemistry solid-state	…	It is not interesting here.
Quantum ESPRESSO	https://www.quantum-espresso.org/	Quantum ESPRESSO is an integrated suite of Open-Source computer codes for electronic-structure calculations and materials modeling at the nanoscale. It is based on density-functional theory, plane waves, and pseudopotentials.
Quentic Demo	https://www.quentic.com/software/hazardous-chemicals/exposure-to-chemical-substances/	Quentic is a system to document and report exposure to CMR substances that being, hazardous chemicals with carcinogenic, mutagenic, or reprotoxic properties, can pose significant health risks.
QUEST	https://teale.chem.nottingham.ac.uk/page/	QUantum Electronic Structure Techniques (QUEST) is our in-house development platform. The code is written predominantly in python, allowing for rapid implementation and prototyping of new quantum chemical methods.
QuteMol	http://qutemol.sourceforge.net/	Molecular visualization-representation. QuteMol is an open source (GPL), interactive, high quality molecular visualization system. QuteMol exploits the current GPU capabilities through OpenGL shades to offers an array of innovative visual effects.
Random coil index		It is not interesting here.

RasMol	http://www.openrasmol.org/	RasMol is a program for molecular graphics visualization originally developed by Roger Sayle. This site is provided for the convenience of users of RasMol.
Raster3D	http://skuld.bmsc.washington.edu/raster3d/raster3d.html	Raster3D is a set of tools for generating high quality raster images of proteins or other molecules.
RasTop	https://www.geneinfinity.org/rastop/	RasTop is a molecular visualization software adapted from the program RasMol. It is particularly adapted for educational purposes and for the analysis of macromolecules at the bench.
RCSB MBT Viewers	https://biojava.org/wiki/RCSB_Viewers:About	Molecular visualization. The RCSB Viewers suite of frameworks provides the capability of creating 3-D viewing applications.
Ribbons	https://web.chemdoodle.com/demos/pdb-ribbons	Visualization of proteins.
RINalyzer	https://rinalyzer.de/	Protein visualization. RINalyzer provides a number of important methods for analyzing and visualizing residue interaction networks (RINs). A RIN is constructed from the 3D structure of a protein as stored in PDB files from the Protein Data Bank
RmscopII		Obsolete.
Rpluto	https://www.ccdc.cam.ac.uk/support-and-resources/support/case/?caseid=de8990ea-5675-4763-8335-39129dd0bf39	Crystallography. RPluto is a tool for visualizing molecular crystal structures.
Rubychem		It is not interesting here.
SAMSON	https://www.samson-connect.net/	Molecular visualization, virtual reality, molecular dynamics. SAMSON is the platform for integrated molecular design. SAMSON's goal is to design drugs, materials and nanosystems.
Scigress	https://www.fqs.pl/en/chemistry/products/scigress	Molecular visualization. SCIGRESS is a multiplatform molecular design, modeling and dynamics software suite designed with experimental chemists. A suite of molecular builders and visualization tools enable the researcher to build novel structures.
Scilab	…	Obsolete.
sCULPT	…	Obsolete.
Setor	…	Obsolete.
Shelxle	https://www.shelxle.org/shelx/eingabe.php	ShelXle combines an editor with syntax highlighting for the SHELXL-associated .ins (input) and .res (output) files with an interactive graphical display for visualization of a three-dimensional structure including the electron density (Fo) and difference density (Fo-Fc) maps.
SHIFTCOR RMN	…	It is not interesting here.
SIESTA	https://departments.icmab.es/leem/siesta/	SIESTA is both a method and its computer program implementation, to perform efficient electronic structure calculations and ab initio molecular dynamics simulations of molecules and solids.
Spartan	https://www.wavefun.com/	Spartan is a molecular modelling and computational chemistry application from Wavefunction.
Spatial Discrete Event Simulat.	…	It is not interesting here.
STRUCTURELAB	…	Obsolete.
Swiss PDB viewer	https://spdbv.vital-it.ch/	Swiss-PdbViewer (aka DeepView) is an application that provides a user friendly interface allowing to analyze several proteins at the same time. The proteins can be superimposed in order to deduce structural alignments and compare their active sites or any other relevant parts.
SYBYL	http://sitios.csic.es/web/calculo-cientifico/sybyl	Software for the modeling and simulation of organic molecules and drug design.
Sybyl-X	https://www.certara.com/pressrelease/certara-enhances-sybyl-x-drug-design-and-discovery-software-suite/	SYBYL-X is a comprehensive suite of computer-aided design tools developed to expedite drug design and other molecular discovery projects, from high throughput screening to late lead optimization.
TeraChem	http://www.petachem.com/products.html	TeraChem is general purpose quantum chemistry software.
Tessel Fortran	…	Obsolete.
Tinker Molecular Modeling	https://dasher.wustl.edu/tinker/	Molecular mechanics and dynamics calculations, with some special features for biopolymers.
Turbomole	https://www.turbomole.org/	Quantum chemistry.
UCSF	https://www.cgl.ucsf.edu/chimera/	UCSF Chimera is a program for the interactive visualization and analysis of molecular structures and related data, including density maps, trajectories, and sequence alignments. It is available free of charge for noncommercial use.
Ugene	http://ugene.net/	The 3D Structure Viewer is intended for visualization of 3D structures of biological molecules.
UHBD obsolete	…	Obsolete.
UniChem	…	It is not interesting here.
Valence bond programs	…	It is not interesting here.

VASPMO	https://sourceforge.net/projects/vaspmo	VASPMO aims at visualizing wavefunctions (or molecular orbitals) from VASP calculations. It reads VASP's output files PROCAR and CONTCAR, and exports an *.out file in Gaussian's output format, which can be visualized by many popular visualization tools, such as JMol, Molekel, Chemcraft, Gabedit and Molden, etc. These tools can further generate *.cube files, which can be visualized by an even wider range of softwares.
VegaHub	https://www.vegahub.eu/	Modelling, 3D visualization. VEGA platform allows access a series of QSAR (quantitative structure-activity relationship) models for regulatory purposes, or develop your own model for research purposes.
Vibeplot	https://sourceforge.net/projects/vibeplot/	Vibeplot presents a way to visualize vibrational analysis from density functional calculations (DFT).
VIDA	https://www.eyesopen.com/vida	A tool to study the data. It is about understanding, rationalizing, predicting, and, most importantly, communicating results.
Viewmol	http://viewmol.sourceforge.net/	It is able to graphically aid in the generation of molecular structures for computations and to visualize their results
Vienna Ab initio Simulation	https://www.vasp.at/	Vienna Ab initio Simulation Package (VASP) is a computer program for atomic scale materials modelling, e.g. electronic structure calculations and quantum-mechanical molecular dynamics, from first principles.
ViewMol	…	Obsolete.
Virtual Chemistry 3D	https://vchem3d.univ-tlse3.fr/	Interactive 3D animations and structures, with supporting information for some topics covered during an undergraduate chemistry degree.
VisualMolecularDynamic VMD	http://www.ks.uiuc.edu/Research/vmd/	VMD is designed for modeling, visualization, and analysis of biological systems such as proteins, nucleic acids, lipid bilayer assemblies, etc. It may be used to view more general molecules.
WebLab	https://www.scalacs.org/TeacherResources/	WebLab Viewer Lite software is being provided "as is" to teachers in Grades Kindergarten through 12 (K-12).
What If	…	It is not interesting here.
WIEN2k	…	It is not interesting here.
XDrawChem	https://www.woodsidelabs.com/chemistry/xdrawchem.php	2D software.
XMol	http://www.hpc.unm.edu/~chem/xmol/xmol.html	Obsolete.
X-PLOR	…	Obsolete.
XtalView	…	Obsolete.
XyMTeX	…	It is not interesting here.
YAMBO code	http://www.yambo-code.org/	YAMBO is an open-source code released within the GPL. It implements Many-Body Perturbation Theory (MBPT) methods (such as GW and BSE) and Time-Dependent Density Functional Theory (TDDFT), which allows for accurate prediction of fundamental properties as band gaps of semiconductors, band alignments, defect quasi-particle energies, optics and out-of-equilibrium properties of materials.
Yasara	http://www.yasara.org/	YASARA is a molecular-graphics, -modeling and -simulation program.
Zeus	http://www.al-nasir.com/portfolio/zeus/	Zeus molecular visualization software.
Zindo	…	It is not interesting here.
